# Evidence linking calcium to increased organo-mineral association in soils

**DOI:** 10.1007/s10533-021-00779-7

**Published:** 2021-04-04

**Authors:** Mike C. Rowley, Stephanie Grand, Jorge E. Spangenberg, Eric P. Verrecchia

**Affiliations:** 1 Institut des dynamiques de la surface terrestre (IDYST), Université de Lausanne, Lausanne, Switzerland; 2 Energy and Geosciences Division, Earth and Environmental Sciences Area, Lawrence Berkeley National Laboratory, Berkeley, CA USA

**Keywords:** Soil organic carbon, Density fractionation, Carbon stable isotopes, X-ray photoelectron spectroscopy, Rock-Eval® pyrolysis

## Abstract

Geochemical indicators are emerging as important predictors of soil organic carbon (SOC) dynamics, but evidence concerning the role of calcium (Ca) is scarce. This study investigates the role of Ca prevalence in SOC accumulation by comparing otherwise similar sites with (CaCO_3_-bearing) or without carbonates (CaCO_3_-free). We measured the SOC content and indicators of organic matter quality (C stable isotope composition, expressed as *δ*^13^C values, and thermal stability) in bulk soil samples. We then used sequential sonication and density fractionation (DF) to separate two occluded pools from free and mineral-associated SOC. The SOC content, mass, and *δ*^13^C values were determined in all the fractions. X-ray photoelectron spectroscopy was used to investigate the surface chemistry of selected fractions. Our hypothesis was that occlusion would be more prevalent at the CaCO_3_-bearing site due to the influence of Ca on aggregation, inhibiting oxidative transformation, and preserving lower *δ*^13^C values. Bulk SOC content was twice as high in the CaCO_3_-bearing profiles, which also had lower bulk *δ*^13^C values, and more occluded SOC. Yet, contrary to our hypothesis, occlusion only accounted for a small proportion of total SOC (< 10%). Instead, it was the heavy fraction (HF), containing mineral-associated organic C, which accounted for the majority of total SOC and for the lower bulk *δ*^13^C values. Overall, an increased Ca prevalence was associated with a near-doubling of mineral-associated SOC content. Future investigations should now aim to isolate Ca-mediated complexation processes that increase organo-mineral association and preserve organic matter with lower *δ*^13^C values.

**Supplementary Information:**

The online version of this article (10.1007/s10533-021-00779-7) contains supplementary material, which is available to authorized users.

## Introduction

Soil geochemical properties are emerging as important predictors of soil organic carbon (SOC) accumulation and content (Blankinship et al. 2018). Yet empirical data on the processes driving this relationship are still scarce. It is well established that iron (Fe) and aluminium (Al) forms can stabilise SOC, leading to its accumulation in soils (Kögel-Knabner et al. 2008; Torn et al. 1997). Yet, calcium (Ca) forms can also play an important role in SOC accumulation (Boiteau et al. 2020; Martí-Roura et al. 2019; Oades 1988), mediating its stabilisation through several potential mechanisms (Rowley et al. 2018). Calcium is thought to indirectly contribute to the accumulation of occluded SOC through the promotion of aggregation (Muneer and Oades 1989b; Oades 1984, 1988) and to the accumulation of mineral-associated SOC through cation bridging processes (Edwards and Bremner 1967; Kalinichev and Kirkpatrick 2007; Sutton et al. 2005). However, very few studies have attempted to quantify the role of these separate processes in the accumulation of organic C in soils with a varied Ca content.

To investigate the processes that cause SOC to accumulate, bulk soil samples may be separated into physical pools through size or density fractionation (DF). Yet, due in part to methodological difficulties arising from the common presence of inorganic C in Ca-rich samples (Rovira et al. 1998), relatively few fractionation studies have focused on the role of Ca in SOC accumulation. Several studies have used DF on Ca-rich soils (Schrumpf et al. 2013; Vormstein et al. 2020; Wen et al. 2017), but fundamental uncertainties remain regarding the mechanisms that govern SOC accumulation in soils with a varied Ca prevalence (Rowley et al. 2018). These processes could be analysed by evaluating how SOC pools vary in otherwise similar soils with either a large or limited prevalence of Ca.

Differences in SOC dynamics can lead to changes in the properties or quality of organic matter (OM) within a soil profile. In particular, the C stable isotope composition (*δ*^13^C values) of OM can be used to investigate the transformation of SOC in different pools or fractions. The *δ*^13^C values of SOC typically increase by approximately 1–3 ‰ with depth, which has been linked to fractionation during oxidative microbial transformation processes (Boström et al. 2007; Hasinger et al. 2015; Hobbie et al. 1999). Variations in *δ*^13^C values between different soils have also been reported, which could not be ascribed to differences in vegetation, and thus, are most likely explained by differences in OM transformation processes. Minick et al. (2017) demonstrated that bulk *δ*^13^C values were lower in soils after Ca-addition (CaSiO_3_), relative to control soils with a limited Ca prevalence. Increased aggregation, driven by the flocculation of soil separates by Ca^2+^ (Muneer and Oades 1989b), could occlude and physically protect SOC from oxidative microbial transformation processes; thereby inhibiting fractionation and preserving low *δ*^13^C values (Minick et al. 2017). However, this hypothesis still requires empirical confirmation.

The thermal stability of OM has also been proposed as a proxy for SOC dynamics (Plante et al. 2009; Sanderman and Grandy 2020). Among thermal analysis techniques, Rock-Eval® analysis can provide insights into OM quality changes (Disnar et al. 2003; Matteodo et al. 2018; Poeplau et al. 2017). In particular, the *I* and *R Index* scores, calculated from the amounts of hydrocarbon compounds released during pyrolysis, have been proposed as an indicator of variations in the OM quality of diverse soils (Sebag et al. 2006; Sebag et al. 2016). In a study covering a range of geochemically diverse soil types across the Swiss Alps, Matteodo et al. (2018) discovered that CaCO_3_-bearing profiles typically had lower *R Index* scores (lower thermal stability). Yet, more investigation into the effects of Ca prevalence on the thermostability of SOC are still needed as a recent study conversely demonstrated that Ca-addition increased the thermal stability of model C substrates (Barreto et al. 2020). These measures of OM quality could also be coupled with surface-sensitive analyses such as X-ray photoelectron spectroscopy (XPS), to yield complementary information on the oxidative transformation of SOC and its interactions with other elements. Yet, to our knowledge these complementary techniques have not been combined to investigate the effects of a varied Ca prevalence on OM quality in different SOC pools.

To investigate the influence of Ca prevalence on SOC accumulation mechanisms, three profiles from a CaCO_3_-bearing and a CaCO_3_-free site, which had developed under similar soil forming conditions (Rowley et al. 2020), were fractioned by sequential sonication and density separation. Samples were split into four fractions (a free-light fraction, two occluded-light fractions, and a heavy fraction) to investigate whether SOC was predominantly stored within free particulate OM, aggregates of increasing sonication resistance (assumed to represent aggregates of differing tensile strength), or mineral-association, respectively. We measured the SOC content, *δ*^13^C values, and Rock-Eval® thermal signature of bulk (unfractionated) soil samples. We also quantified the SOC content, mass, and *δ*^13^C values in all the fractions. Finally, we measured a subset of fractions with XPS to characterise the surface chemistry of our samples. Our guiding hypothesis was that the flocculation of soil separates by Ca^2+^ would cause an accumulation of occluded SOC at the CaCO_3_-bearing site. Furthermore, we hypothesised that this occlusion would inhibit microbially-driven oxidative transformation of SOC and its associated C isotope fractionation, resulting in lower bulk *δ*^13^C values. Overall, we found that occlusion played a minimal role in SOC dynamics at either site (accounting for < 10% of total organic C). It was instead the mineral-associated fraction that explained the two-fold difference in SOC content and the divergence in bulk *δ*^13^C values.

## Materials and methods

### Site description and sampling

This study was completed in the Nant Valley (573′000, 119′000, CH1903 LV03), Vaud Alps, Switzerland. The Valley is situated on the Morcles Nappe, which is a tectonic unit consisting of Jurassic and Cretaceous shallow-water limestones intercalated with marl and shale deposits (Austin et al. 2008). Sampling took place in the rangeland (Suppl. Fig. S1) described in detail by several studies (Ceperley et al. 2020; Grand et al. 2016; Rowley et al. 2020; Vittoz and Gmür 2008). The rangeland is 1500 m above sea level, receives approximately 1800 mm year^−1^ precipitation, and has a mean annual temperature of 6 °C (Vittoz and Gmür 2008).

Two sampling sites were selected at the rangeland. We dug three profiles at each site (Suppl. Fig. S1), which were characterised (IUSS Working Group WRB 2015) as being either Eutric Cambisols (siltic) with no CaCO_3_ (CaCO_3_-free; F1, F2, F3) or Cambic Phaeozems (siltic) with a small (< 6.2%) CaCO_3_ content (CaCO_3_-bearing; B1, B2, B3). Profiles were sampled at 6–7 depth intervals and labelled from 1 to 6/7 with increasing depth (e.g., F1.1-to-F1.6). The texture, silicate mineralogy, and elemental composition of the profiles were highly similar except for an increased relative abundance of Ca in the Cambic Phaeozems (see Rowley et al. 2020 for details). Both above- and below-ground biomass (AGB and BGB) were also randomly sampled at both sites to assess potential variations in the *δ*^13^C values of vegetation.

### Sample preparation

Samples of AGB and BGB were oven-dried (65 °C, to constant weight) and ground by hand. Air-dried soil samples were sieved to 2 mm prior to density fractionation. A subsample of each bulk soil sample was ground in a rotary mill for SOC elemental and isotope analyses (Rowley et al. 2020). Prior to these analyses, inorganic C was removed through a HCl fumigation procedure and a correction factor was applied to account for changes in mass (Harris et al. 2001).

### Laboratory analysis

Quality control procedures included the analysis of an internal standard when appropriate, as well as the inclusion of blanks and quality checks.

#### Density fractionation

**Fig. 1:**
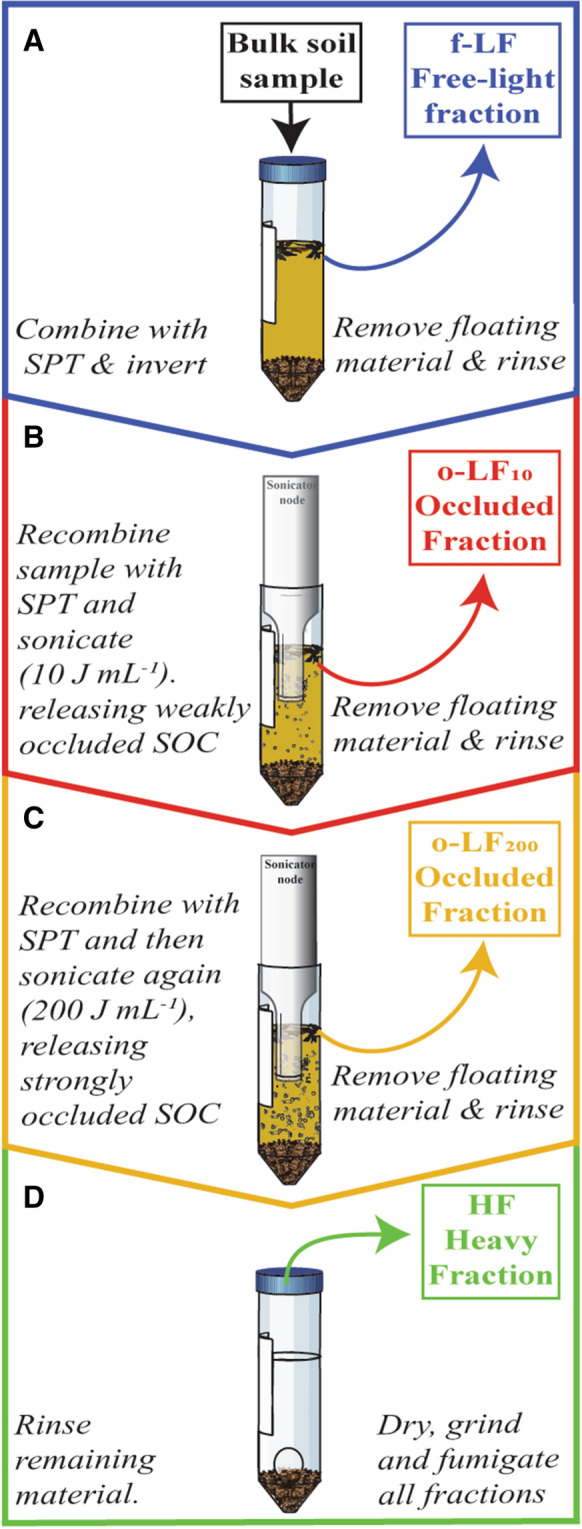
Schematic showing the sequential sonication and density separation process. **a** The free-light fraction (f-LF) was first fractionated from the bulk soil by inverting the sample. **b** The first occluded light fraction (o-LF_10_) was then separated after 10 J mL^−1^ sonication. **c** The second occluded light fraction (o-LF_200_) was then separated after 200 J mL^−1^ sonication. **d** The remaining mineral-associated material then forms the heavy fraction (HF). All fractions were thoroughly rinsed prior to analysis.

Soil samples were fractioned into four SOC pools (Fig. 1a, d) using sequential sonication and density separation (Golchin et al. 1994; Poeplau et al. 2018; Viret and Grand 2019). A 7 g soil aliquot was combined with 35 mL 1.6 g cm^−3^ sodium polytungstate (SPT) in 50 mL centrifuge tubes and inverted 10 times by hand to liberate the f-LF. Samples were then left to settle for 30 min before centrifuging (1080 g for 30 min) to separate the floating free-light fractions (f-LF) from the remaining sample. The floating f-LF were decanted onto 0.45 μm nitrocellulose membranes and vacuum filtered. The f-LF remaining on the filter were then thoroughly rinsed thoroughly with deionised water (Schrumpf et al. 2013) and washed into aluminium drying boats (Fig. 1a).

Sodium polytungstate was placed back into the tubes and the samples were then sequentially sonicated using a pre-calibrated QSonica Q500 Sonicator with a model cl-334 Sonication Node (see North 1976 and Schmidt et al. 1999 for details on the methods). Sonication energies were selected after pretesting, which revealed that higher sonication energies (up to 590 J mL^−1^; Schmidt et al. 1999; Kaiser and Berhe 2014) did not increase the recovery of occluded material at either site (data not shown; Golchin et al. 1994; Schrumpf et al. 2013). The output energy of the Sonication Node was calibrated calorimetrically according to Schmidt et al. (1999). Tubes were placed in an ice slurry to dissipate heat transferred from the sonicator node, which was run at 20% amplitude. The first sonication was carried out at 10 J mL^−1^, separating the first set of occluded fractions (o-LF_10_) in the same manner as the f-LF (Fig. 1b). The samples were then recombined with the SPT and sonicated a second time to 200 J mL^−1^, prior to separating the floating material in the same manner as the f-LF and o-LF_10_, resulting in the recovery of a second set of occluded fractions (o-LF_200_; Fig. 1c). The remaining heavy fractions (HF; Fig. 1d) were rinsed five times by centrifugation (30 min at 7500×*g*) until conductivity was reduced to < 100 μS (Schrumpf et al. 2013). To ensure effective removal of the SPT, HF centrifuge pellets were disrupted with a 1 mm glass bead on a vortex (Fig. 1d) and then placed on a rotary shaker (10 min at 250 rpm) between rinses (Schrumpf et al. 2013). Once rinsed, all fractions were oven dried at 65 °C and weighed to the nearest mg. Subsamples of the HF were ground in a ball mill, while the LFs were ground to a fine powder by hand. To ensure accurate and replicable fractionation, DF was run in triplicate for each soil sample. Recovery rates ranged from 98 to 100% (Suppl. Table S1). There was no o-LF_10_ recovered from sample F1.6.

The precipitation of Ca-metatungstate on light fraction material in soils with a high Ca prevalence could create a false equivalence between the light fractions (LFs; free and occluded fractions combined) and HF (Rovira et al. 1998). This has been observed in soils with higher quantities of CaCO_3_ (> 40%; Rovira et al. 1998) than our CaCO_3_-bearing site (< 6.2%). Significant precipitation of Ca-metatungstate on our fractions can however be ruled-out because: (i) recovery during DF was not higher at the CaCO_3_-bearing site (Suppl. Table S1), (ii) XPS (methods detailed below) revealed that W contamination was lowest in the HF, with no difference between the sites (Suppl. Table S2), and (iii) there was also no peak detected for Ca-metatungstate (shift towards 35 eV) in the detailed scans of the W_4f_ region (Suppl. Fig. S2). Thus, these differences did not seem to arise due to the precipitation of Ca-metatungstate on fresh particulate OM, but future studies should be aware of the risks of running DF on CaCO_3_-bearing soil samples (> 20%).

#### Soil organic carbon analysis (*δ*^13^C values and Rock-Eval®)

Soil organic carbon content and C isotope compositions of bulk samples, triplicates of density fractions, AGB, and BGB were determined using a Carlo Erba 1108 elemental analyser connected to a Thermo Fisher Delta V isotope-ratio mass spectrometer (EA/IRMS system from Bremen, Germany). The EA/IRMS was operated in continuous He flow mode via a split interface (Conflo II). Combustion of samples occurred within pre-weighted Sn capsules in an O_2_ atmosphere at 1020 °C. The carbon isotope compositions were expressed in the delta (*δ*) notation as the per mil (‰) difference of the ^13^C/^12^C ratio in the sample relative to the Vienna Pee Dee Belemnite standard (*δ*^13^C in ‰ VPDB; Coplen 2011). A 3-point calibration with international reference materials and in-house standards was used to calibrate and normalise the isotopic ratios to the international scale (VPDB-LSPVEC lithium carbonate). The intermediate precision and accuracy of the EA/IRMS analyses was assessed through replicate analyses of separate reference materials and was better than 0.05 ‰. N measurements were not established in this study with the EA/IRMS, but bulk values, established on a different elemental analyser can be found in Rowley et al. (2020). Percentages are presented on a mass basis. The mass of SOC in the different fractions was calculated by multiplying the SOC content by the quantity of material recovered in each fraction.

The thermal stability of OM in ground and non-fumigated bulk-soil samples was also measured with a Rock-Eval® 6 Pyrolyser (Vinci Technologies, Rueil-Malmaison, France). Full details on the Rock-Eval® methods and different indices can be found in the supplementary information, "Materials and methods". The S2 thermogram was split into 5 separate components (A1:A5) at fixed temperature bounds and then used to calculate the *I* and *R Index* scores according to Eqs. 1 and 2 (Malou et al. 2020; Sebag et al. 2016). The *I* and *R Index* scores from our samples were then compared to the negative linear trend (“humic” trend) from geochemically- (Matteodo et al. 2018) and pedoclimatically-diverse datasets (Sebag et al. 2016). This trend in thermal stability is commonly ascribed to changes in OM quality upon decomposition in soils (Malou et al. 2020; Thoumazeau et al. 2020).

1 $$I= {\mathrm{log}}_{10}\left(\frac{\left(\mathrm{A}1+\mathrm{A}2\right)}{\mathrm{A}3}\right)$$

2 $$R=\frac{\mathrm{A}3+\mathrm{A}4+\mathrm{A}5}{100}$$

#### X-ray photoelectron spectroscopy

All the fractions of a surface and subsoil sample from a randomly selected profile at each site were measured (B2.1 to B2.4; F2.1 to F2.4) using a PHI VersaProbe II Scanning XPS Microprobe (Physical Instruments AG, Feldkirchen, Germany). Measurements with the XPS were performed at the Surface Characterization Laboratory, *Ecole Polytechnique Fédérale de Lausanne*. Sample topography can influence XPS measurements due to differences in photoelectron emission geometry (Zemek et al. 2008). Thus, powdered fractions were loaded onto stubs in a homogeneous manner. The surface of samples (< 10 nm depth; Yuan et al. 1998) was then analysed with a monochromatic Al Kα X-ray source (1486.6 eV) with a beam size of 200 µm at 45.7 W. The spherical capacitor was set at 45° take-off angle respective to the surface of samples. Samples were scanned twice, once coarsely (regional scans), with a pass energy of 187.9 eV, which yielded the principal elements of interest. The samples were then scanned again in more detail (survey scans) using a pass energy of 47 eV to investigate the identified surficial elements. Exposure time was < 30 min to prevent X-ray induced alteration of the density fractions and subsequent false C assignments (Dengis et al. 1995). Vacuum inside the main chamber was in low 10 torr during measurements (− 7 Pa). Sample charging during analysis caused peak shifts of < 3 eV, which were corrected based on the maximum principal C_1s_ peak, centred at 285 eV (Mikutta et al. 2009).

Atomic quantification of the surface of samples was completed using a process of background linear subtraction, fitting a set of Gaussian curves to spectra and converting intensities into atomic abundancies with sensitivity factors (Moulder and Chastain 1992). Curve fitting of survey scans was performed using PHI Multipak 9.5™ Software. Identification of binding energies was completed according to Moulder and Chastain (1992). Spectral shifts in core level C_1s_ binding energies were assigned according to Table 1, deconvoluting the C_1s_ peak into sub-peaks that are indicative of different C bonding environments (Suppl. Fig. S4; Jones and Singh 2014). Sub-peaks were fitted with Gaussian-Lorentzian functions, the full-width-at-half-maximum was allowed to vary between 1 and 2. The ratio of aliphatic / aromatic C to oxidised C moieties (alcoholic/phenolic, carbonyl, carboxylate groups) was used to quantify the degree of oxidative transformation of surficial C (Yeasmin et al. 2017).

**Table 1:** Binding energies of specific carbon C_1s_ sub-peaks and their associated C bonding environment

Associated carbon bond environment	Bond type	Fixed binding energy (eV)
Aliphatic/aromatic	C–H/C–C	285
Alcoholic/phenolic	C–OH	286.5
Carbonyl	C=O	288
Carboxylate	O=C–OH	289.5

The figures below have been adapted from Jones and Singh (2014) and Moulder and Chastain (1992)

### Statistical analysis

The effects of the presence or absence of CaCO_3_ on SOC distribution and *δ*^13^C values were investigated using linear mixed models. Models were fitted using SAS 9.4™. The estimation method was set to restricted (residual) maximum likelihood. Residuals were checked for goodness of fit and normality with quantile-quantile plots (Galecki and Burzykowski 2015). Deviations from homoscedasticity were evaluated by plotting conditional residuals against predicted values. The significance of fixed effects was evaluated using type III F-tests. The Satterthwaite adjustment was used to compute the degrees of freedom of the denominators (Satterthwaite 1946). Comparison of the means of significant variables were completed using t-tests without multiple inference adjustment (Webster 2007). The alpha level (α) of significance was set at 0.05. All reported means in "Results" are conditional least-square means ± standard error of the mean.

Separate models were constructed for the analysis of bulk soil and DF measurements. Simpler model structures were used for bulk observations because they were based upon singular rather than triplicate measurements. Full details on the model structures used for bulk observations can be found in the supplementary information, "Materials and methods". Models that were used to analyse DF triplicate observations included site (CaCO_3_-free or CaCO_3_-bearing), classes of sample depth (0–5 cm, 5–10 cm, etc.…), fractions (f-LF, o-LF_10_, o-LF_200_, and HF), and their interactions as fixed effects. Depth was set as a random effect with a first-order autoregressive covariance structure, while variance estimates were permitted to vary between sites. Rather than using the mean of triplicate observations as the response variable, each observation was accounted for separately. This was achieved by setting each fraction as a repeated measure with a variance component covariance structure. Variance estimates were also allowed to vary between the LFs or the HF to account for data heteroscedasticity.

## Results

### Bulk soil

**Fig. 2:**
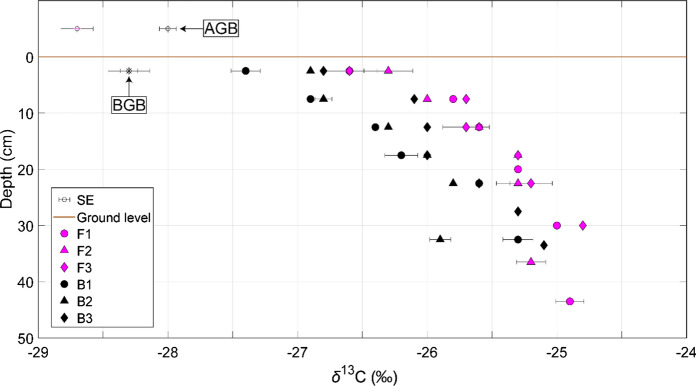
Carbon isotope compositions (*δ*^13^C values, ‰ vs. VPDB) of bulk soil organic carbon, above-ground (AGB) and below-ground biomass (BGB) from the CaCO_3_-free (F in fuchsia) and CaCO_3_-bearing (B in black) site. Error bars represent the standard error of the mean (SE) of duplicate measurements

The SOC content in bulk samples (unfractionated) was twice as high at the CaCO_3_-bearing site (Suppl. Table S3). Bulk *δ*^13^C values increased systematically with depth at both sites. As hypothesised, bulk *δ*^13^C values were lower at the CaCO_3_-bearing site relative to the CaCO_3_-free site, with an average offset of approximately 0.8 ‰. The *δ*^13^C values of AGB were lower at the CaCO_3_-free site, but BGB *δ*^13^C values were indistinguishable between the sites (Fig. 2).

**Fig. 3:**
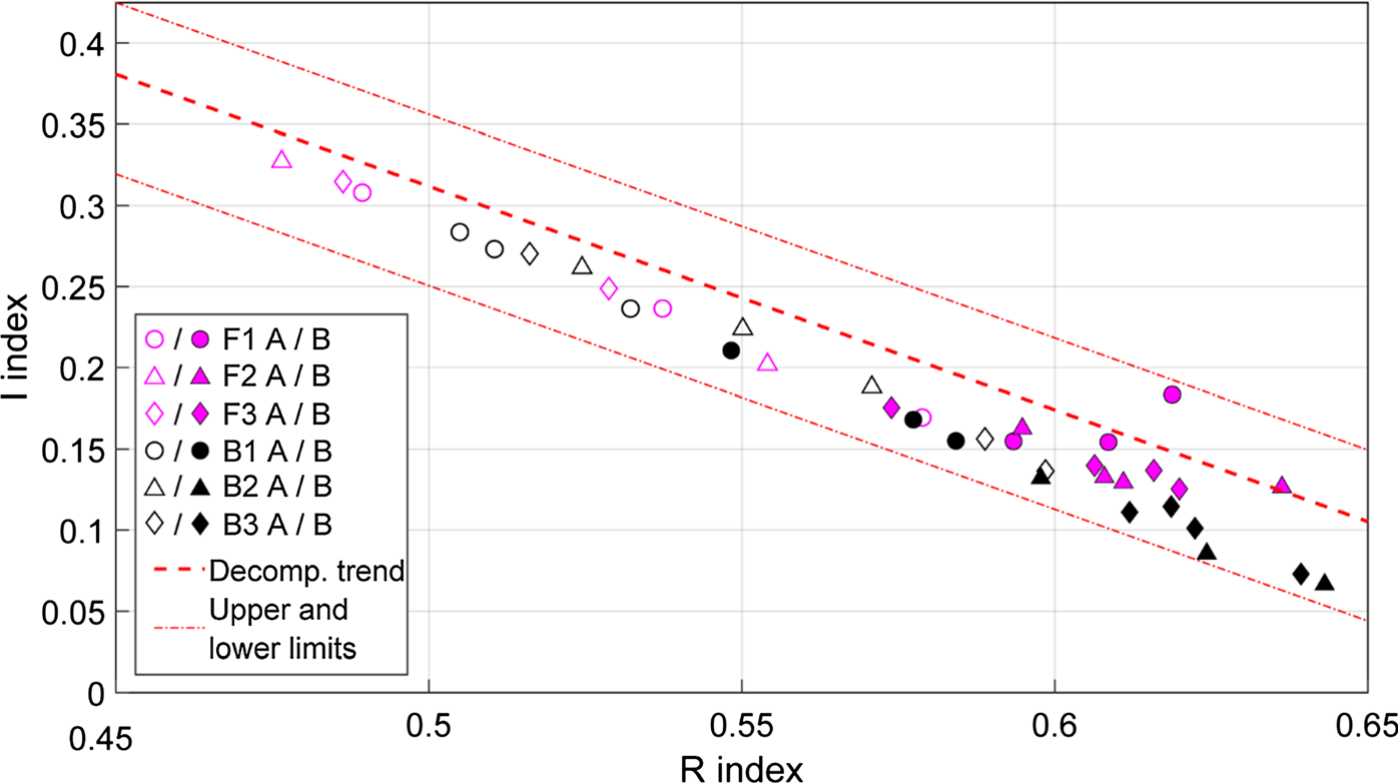
Rock-Eval^®^ *I* and *R Index* scores of bulk soil from the CaCO_3_-free (in fuchsia) and CaCO_3_-bearing (in black) profiles, split by horizon (A horizon with hollow symbol/B horizon with filled symbol). Both the linear decomposition (Decomp.) trend (“humic” trend) and upper/lower boundaries of the range of reported *I* and *R index* scores, seen in a geochemically- and pedoclimatically diverse dataset, have been plotted as reference lines in red (see Sebag et al. 2016 for more details). See Suppl. Fig. 6 for the direct comparison of results from this study with data from other Swiss soils (Matteodo et al. 2018)

The Rock-Eval® results for bulk samples were well within the bounds of usual *I* and *R Index* scores, falling just below the typical decomposition trend (Fig. 3; "humic" trend of Sebag et al. 2016). In the B horizons, the CaCO_3_-free profiles had slightly higher *I Index* scores (Suppl. Table S3). The CaCO_3_-free samples also had a higher proportion of A5 contribution to the S2 thermogram (pyrolysis curve; Suppl. Fig. S5), ensuring that their *R Index* scores remained approximately equivalent to that of the CaCO_3_-bearing samples. Thus, B horizons of CaCO_3_-free profiles had an S2 thermogram that was more-distributed compared to their CaCO_3_-bearing counterparts, with both high (A5) and low-temperature (A1) pyrolysis products being relatively abundant.

### Density fractions

#### Distribution of material and SOC between fractions

The proportion of sample mass in a fraction refers to the amount of material recovered in a specific fraction, relative to the original bulk sample mass. At both sites, the largest proportion of sample mass was recovered in the HF (Suppl. Table S1) and the smallest proportion of sample mass was recovered in the o-LF_10_ (Suppl. Fig. S7a, d). The proportion of sample mass recovered in the occluded light fractions was higher at the CaCO_3_-bearing site.

SOC content refers to the concentration of organic carbon within a sample, where percentages are reported on a mass basis. The SOC content of the HF were similar to the bulk soil at both sites, differing most in surficial horizons (Suppl. Fig. S8): which had a larger proportion of sample mass recovered in the LFs, relative to deeper samples. Consistently with bulk soil results, the SOC content of the HF were higher at the CaCO_3_-bearing site.

**Fig. 4:**
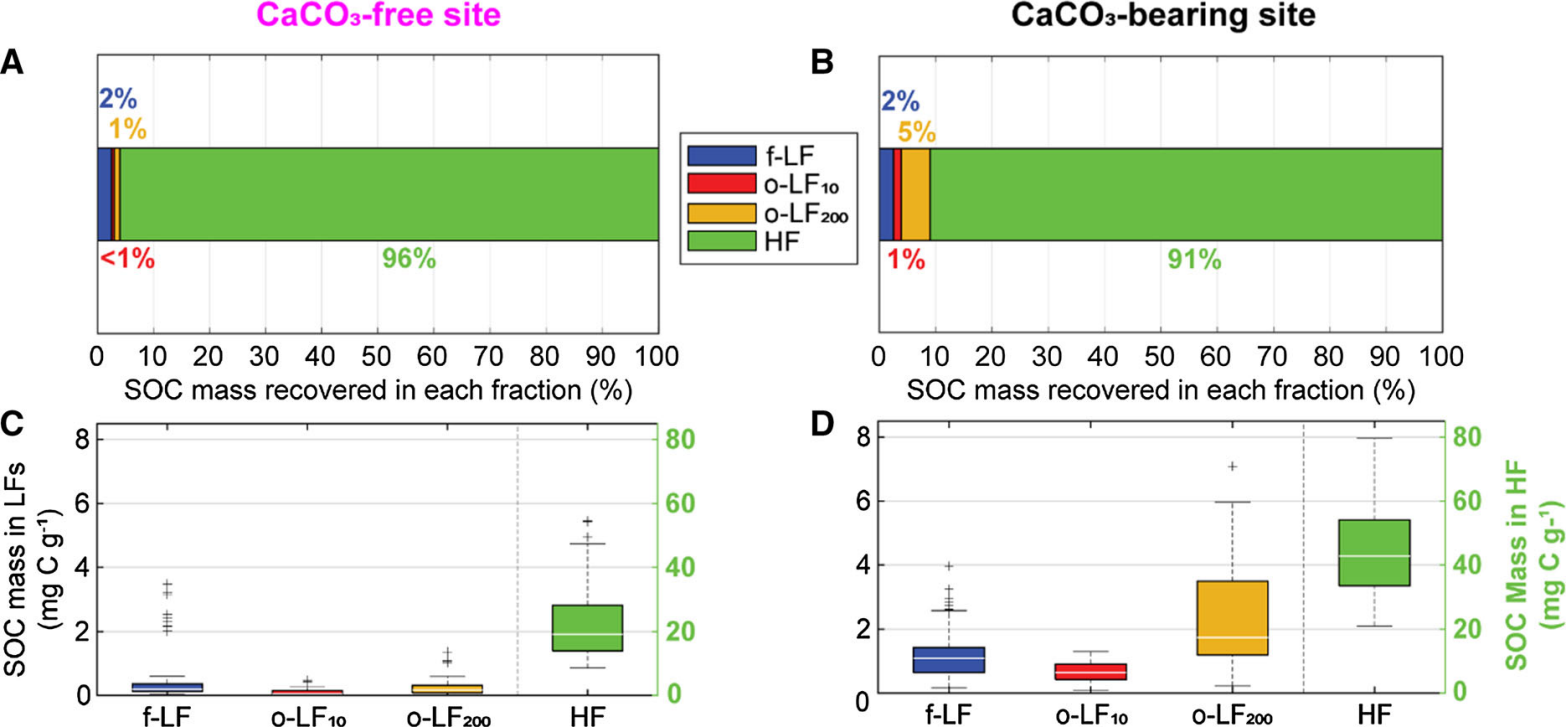
**a** and** b** Average proportion of SOC mass recovered in the fractions (free-light, occluded light fractions separated at 10 and 200 J mL^−1^, and heavy fractions; f-LF, o-LF_10_, o-LF_200_, HF, respectively) from the **a** CaCO_3_-free and **b** CaCO_3_-bearing site as a percent of total SOC mass. **c** and Mass of soil organic carbon (SOC) recovered in the light fractions (LFs; left y axis) and HF (right y axis) from 1 g of oven-dried (105 °C) soil (mg C g^−1^) at the **c** CaCO_3_-free and **d** CaCO_3_-bearing site. Bottom and top edges of the boxes in the box plot represent the 25th and 75th percentiles, the middle bars represent the median. Whiskers represent the range of data points not considered as outliers, while ‘+’ represent values outside of the maximum potential whisker value, corresponding to ± 0.4 SE of the mean (outliers)

The mass of SOC in the different fractions was calculated by multiplying the quantity of material recovered in each fraction by its SOC content. The mass of SOC in the LFs were always at least an order of magnitude lower than the HF at both sites (Fig. 4a, b). The mass of SOC in the LFs were always higher at the CaCO_3_-bearing site than the CaCO_3_-free site (Fig. 4c, d; f-LF = 1.3 ± 0.1 vs. 0.6 ± 0.1 mg C g^−1^; o-LF_10_ = 0.7 ±  0.1 vs*.* 0.1 ± 0.1 mg C g^−1^; o-LF_200_ = 2.5 ± 0.1 vs. 0.3 ± 0.1 mg C g^−1^). The mass of SOC in the HF were also nearly twice as high in the CaCO_3_-bearing site (45.5 ± 0.6 mg C g^−1^), relative to the CaCO_3_-free site (23.1 ± 0.6 mg C g^−1^), demonstrating that soil samples with an increased Ca prevalence contained more mineral-associated SOC.

#### *δ*^13^C values of fractions

**Fig. 5:**
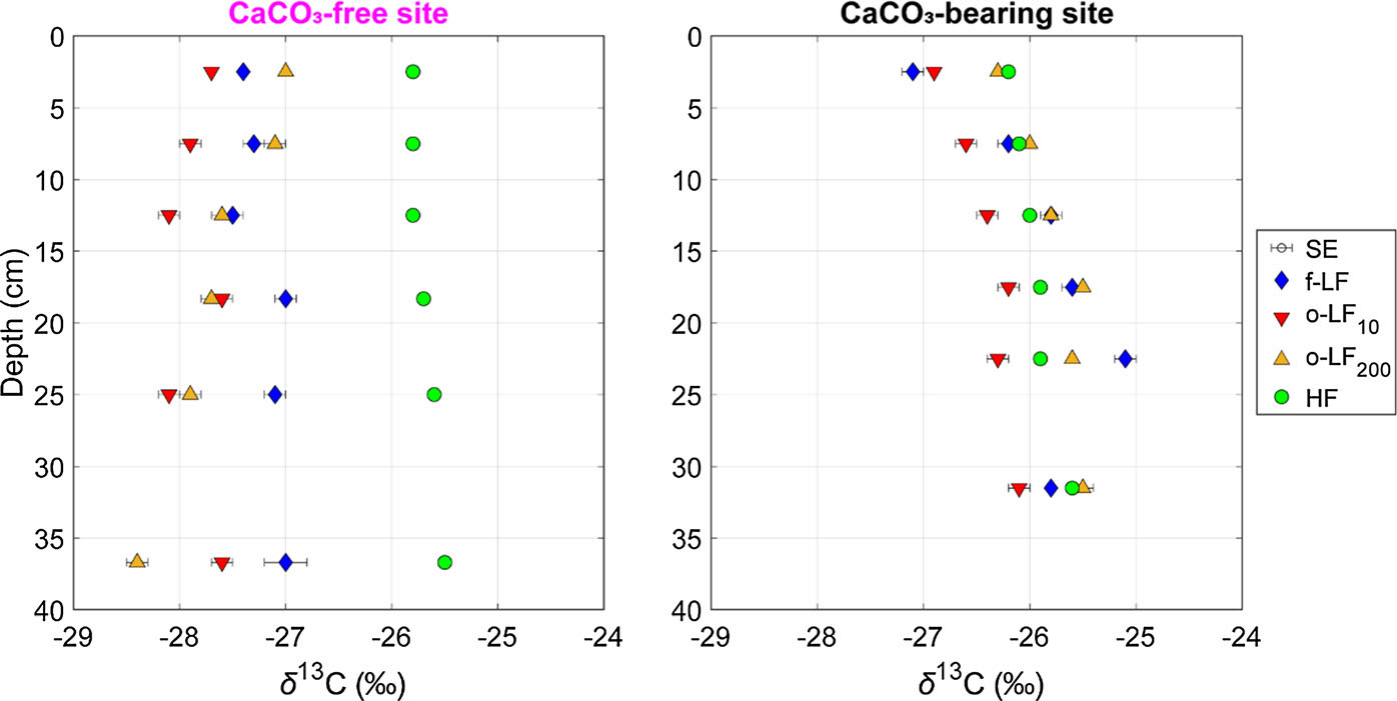
Mean carbon isotope compositions (*δ*^13^C values) of the fractions (free-light, occluded light fractions separated at 10 and 200 J mL^−1^, and heavy fractions; f-LF, o-LF_10_, o-LF_200_, HF, respectively) with sample depth (cm). The symbols represent the mean, the bars the standard error of the mean (SE) of triplicate measurements of the *δ*^13^C values from the CaCO_3_-free (F1, F2, F3) sites on the left and the CaCO_3_-bearing sites (B1, B2, B3) on the right. Depth profiles of *δ*^13^C values for individual profiles can be found in Suppl. Fig. S9

**Fig. 6:**
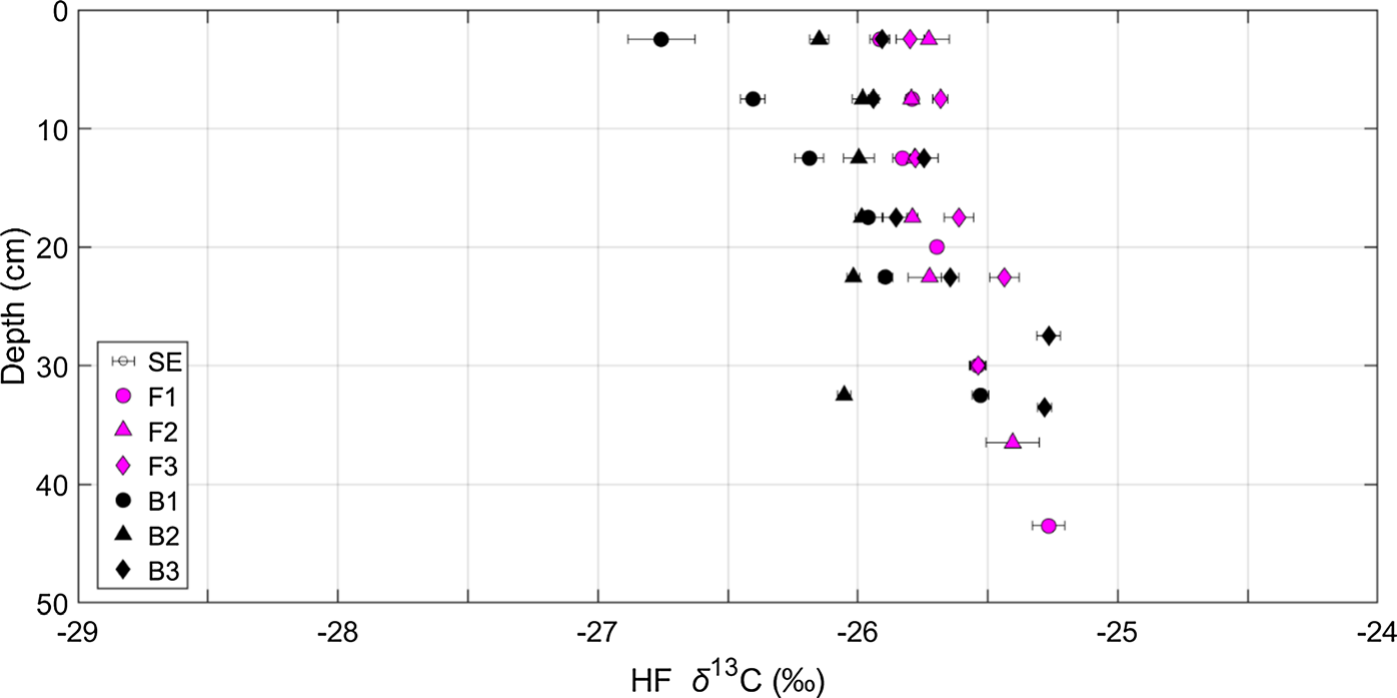
Mean carbon isotope compositions (*δ*^13^C values) of the heavy fractions (HF) from the CaCO_3_-free (F1, F2, F3) and CaCO_3_-bearing (B1, B2, B3) profiles at different depths (cm). Error bars represent the standard error of the mean (SE) of triplicate measurements

The *δ*^13^C values of the LFs at the CaCO_3_-bearing site were always higher than those at the CaCO_3_-free site (f-LF = − 25.9 ± 0.2 ‰ vs. − 27.2 ± 0.1 ‰; o-LF_10_ = − 26.5 ± 0.2 ‰ vs*.* − 27.9 ±  0.1 ‰; o-LF_200_ = − 25.8 ± 0.2 ‰ vs*.* − 27.6 ± 0.1  , respectively; Fig. 5; Suppl. Fig. S9). Yet, *δ*^13^C values of the HF were typically lower at the CaCO_3_-bearing site than at the CaCO_3_-free site, which was particularly evident in B1 or B2, but less apparent in B3 (Fig. 6). Thus, the CaCO_3_-free site displayed an increase in *δ*^13^C values from the LFs to the HF, but the *δ*^13^C values of the LFs were similar to the HF at the CaCO_3_-bearing site (Fig. 5).

There were small differences between the *δ*^13^C values of the bulk and HF, with a slight loss of the depth trend, which could in part be explained by the higher proportions of LF material in surficial samples. To check our trends, measurements of *δ*^13^C values in bulk and HF samples were repeated after some weeks (newly prepared elemental analyser reactor). Results showed a slightly higher variance than was observed between our triplicate measurements. Ultimately, this uncertainty reduced the magnitude of variation in the DF *δ*^13^C values with depth, relative to the bulk *δ*^13^C values.

#### XPS characterisation of fractions

##### Surficial chemical compositions

The two main elements detected by XPS were C (44–57 %) and oxygen (O, 30–40 %). The high C contents were likely caused by the adsorption of adventitious C by the samples. As a result, the surficial composition of the fractions were not reliable (Suppl. Table S2). Elements associated with the mineral or organic phases (e.g. N, Ca, Fe, Si, Ti, or Al) were all detected at low contents. Tungsten and chlorine were also detected at low contents, representing residues of SPT used for fractionation and chlorides from HCl-fumigation, respectively.

##### Patterns in XPS survey scans

**Fig. 7:**
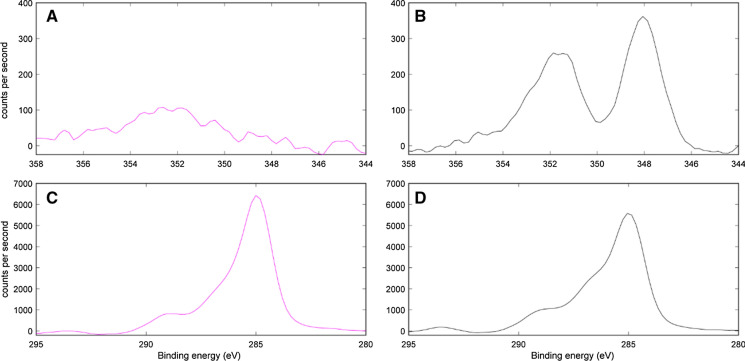
X-ray photoelectron spectroscopy (XPS) spectra in the Ca_2p_ (**a** and **b**) and C_1s_ (**c **and** d**) binding energy (eV) range of the sample subset from the CaCO_3_-free (F2.1 & F2.4; **a **and** c**) and CaCO_3_-bearing (B2.1 & B2.4; **b **and** d**) site. See Suppl. Fig. S10 (Ca_2p_) and Suppl. Fig. S11 (C_1s_) for individual XPS spectra from each fraction

While quantification of the surficial chemical composition of the fractions were unreliable, information could still be drawn from the differences in bonding environments of surficial elements inferred from the XPS scans (Suppl. Fig. S2 & S10–S13). There was a slight shift in the N_1s_ peak towards more protonated N forms at the more acidic CaCO_3_-free site (Suppl. Fig. S13). Calcium metatungstate precipitation was not evident on the details of the W_4f_ scans (Suppl. Fig. S2). There was also a clear difference in the Ca_2p_ signal between the sites (Fig. 7a, b). Both sites presented a peak in the Ca_2P1/2_ region, but this peak was better defined in the CaCO_3_-bearing fractions. Furthermore, the CaCO_3_-bearing site also displayed a satellite peak in the Ca_2P3/2_ region, which was not present at the CaCO_3_-free site.

##### C_1s_ peak deconvolution

The deconvolution of the C_1s_ peak indicated that the largest proportions of surficial C were always associated with aliphatic / aromatic C moieties at both sites (Fig. 7; Table 1; Suppl. Fig. S11). The main difference between our sites was a higher proportion of carbonyl C moieties (288 eV; Fig. 7) in the CaCO_3_-bearing fractions (14 ± 1.1%) than the CaCO_3_-free (9.8 ± 1.3%). There were also noticeable increases in the proportion of aliphatic/aromatic C moieties in the occluded fractions at the CaCO_3_-free site. The ratios of aliphatic/aromatic C to oxidised C moieties in the LFs were typically higher and more similar between fractions at the CaCO_3_-bearing site. Yet, the ratios in the HF were higher at the CaCO_3_-bearing site and decreased with depth.

**Table 2:** Results obtained from the deconvolution of the carbon 1s (C_1s_) X-ray photoelectron spectra for the fractions (free-light, occluded light fractions separated at 10 J mL^−1^ and 200 J mL^−1^, and heavy fractions; f-LF, o-LF_10_, o-LF_200_, HF, respectively) from the CaCO_3_-free (F2.1 & F2.4) and CaCO_3_-bearing (B2.1 & B2.4) sample subset

Sample	Fractions	C–C/C–H	C–O	C=O	O–C=O	Ratio of C–C–C–H to oxidised C (C–O/C=O/O–C=O)
		285 eV	286.5 eV	288 eV	289.5 eV	
F2.1	f-LF	57.6	24.1	13.4	4.9	0.74
	o-LF_10_	73.8	11.6	8.3	6.2	0.35
	o-LF_200_	70.4	16.7	7.6	5.3	0.42
	HF	48.3	29.1	13.2	9.4	1.07
F2.4	f-LF	59.2	23.7	11.2	5.8	0.69
	o-LF_10_	71.7	14.1	7.6	6.5	0.39
	o-LF_200_	70.1	16.5	5.4	8.0	0.43
	HF	64.8	19.6	8.3	7.2	0.54
B2.1	f-LF	61.6	21.0	13.3	4.1	0.62
	o-LF_10_	59.4	22.0	12.8	5.8	0.68
	o-LF_200_	59.1	19.3	15.0	6.7	0.69
	HF	47.5	27.0	17.4	8.1	1.11
B2.4	f-LF	57.6	19.0	16.7	6.7	0.74
	o-LF_10_	63.3	20.3	10.0	6.4	0.58
	o-LF_200_	58.2	22.4	14.8	4.7	0.72
	HF	55.1	25.1	10.8	9.0	0.81

The first four columns represent the percentage area of each sub-peak within the total C_1s_ spectra and are indicative of different C bonding environments (listed in Table 2). The final column represents the ratio between the percentages of the sub-peak centred at 285 eV relative to the percentage representation of other sub-peaks

## Discussion

In this study, we aimed to evaluate the relative importance of different processes in the accumulation of SOC at two otherwise-similar sites, with or without CaCO_3_. Attempting to find soils that had developed under highly similar soil forming conditions with a varied Ca prevalence, we retained only six profiles, which were all in close proximity (< 500 m). More details on the geochemical similarities between these sites can be found in Rowley et al. (2020). Generalisation of the findings from this study to other soils under different environmental conditions is not supported by our experimental layout; yet mechanistic insights gained from this approach can still inform our understanding of SOC dynamics in Ca-bearing soils.

To analyse the processes involved in the accumulation of SOC, we separated free SOC (f-LF) from occluded (o-LF_10_ & o-LF_200_) and mineral-associated SOC (HF) using DF and sequential sonication. We then assume that these specific fractions relate to operationally-defined pools of SOC or processes that store organic C in soils, specifically: f-LF– chiefly plant-derived OM stored without notable interaction with the soil matrix, o-LF_10_––chiefly plant-derived OM trapped within aggregates that have a lower resistance to sonication, o-LF_200_– OM of both plant and microbial origin stored within aggregates that have a higher resistance to sonication, HF– relatively small organic molecules of plant and microbial origin, which are adsorbed onto mineral surfaces. It is still possible that within our HF, there are clay- to nano-sized aggregates that continue to physically protect SOC from further oxidative transformation, contributing to its accumulation (Vogel et al. 2014). However, at this scale, this stabilisation by physical separation is arguably indistinguishable from the influence of sorption, both in terms of biogeochemical signature and conferred stability, so that these processes may be considered as mutually inclusive (See Fig. 1 in Rowley et al. 2018 for more details). We have also made the assumption that increasing sonication energies disrupt aggregates of increasing tensile strength, accessing occluded SOC pools associated with different types of aggregates (see Kaiser and Berhe 2014 for detailed review). While attribution of these occluded fractions to specific aggregate size classes is not possible, we can reasonably assume that, from differences in their tensile strength, material from the o-LF_10_ were stored within aggregates that were more dynamic, relative to the o-LF_200_.

### Ca-mediated occlusion

As hypothesised, the CaCO_3_-bearing site had a larger occluded SOC pool, particularly in the o-LF_200_. This larger pool of occluded SOC could be partially explained by the higher SOC content at the CaCO_3_-bearing site, which is known to positively influence aggregation processes and occlusion (Chenu 1989; Chenu and Cosentino 2011). The presence of CaCO_3_ and an increased Ca content (CaCO_3_-bearing site) has also been repeatedly linked to increased aggregation and a larger pool of occluded SOC (Kaiser et al. 2014; Muneer and Oades 1989a; Paradelo et al. 2016). In humid conditions, this is usually attributed to the flocculation of soil separates by exchangeable Ca^2+^ (Muneer and Oades 1989b), rather than the cementation of aggregates by CaCO_3_ during its evaporative precipitation (Fernández-Ugalde et al. 2014). Thus, the increased occluded SOC pool at the CaCO_3_-bearing site was most likely driven by a mixture of biotic (SOC content and microorganism activity) and abiotic (flocculation by exchangeable Ca^2+^) positive influences on aggregation.

Yet, contrary to our hypothesis, the f-LF and occluded pools were of little overall significance to bulk SOC dynamics at either site. Our results indicated that occluded SOC accounted for between 1–3 % and 3–10 % of total SOC at the CaCO_3_-free and CaCO_3_-bearing site, respectively. We had also expected that the CaCO_3_-bearing site may have had a larger proportion of aggregates with a higher tensile strength (o-LF_200_) in the B horizon, but there were no significant differences in the ratio of o-LF_10_ to o-LF_200_ between our sites. Contrastingly, several key studies have repeatedly highlighted the importance of occlusion mediated by CaCO_3_ to the accumulation of SOC in environments with a low Aridity Index (Blanco-Moure et al. 2012a; Blanco-Moure et al. 2012b; Fernández-Ugalde et al. 2014; Fernández-Ugalde et al. 2011; Virto et al. 2013). The humid conditions present at the Nant Valley could have reduced the importance of the occluded pool and aggregate tensile strength at the CaCO_3_-bearing site, by inhibiting the evaporative precipitation of CaCO_3_ and its cementation of aggregates. Accordingly, using fractionation parameters comparable to ours (sonicated at 300–450 J mL^−1^ for soils with a loamy texture and density cut-off of 1.6 g cm^-3^), Schrumpf et al. (2013) demonstrated that, over a larger range of humid soils, the occluded pool also accounted for a small proportion of total SOC (4–17 %).

A higher sonication energy could have been expected to slightly increase the recovery in our occluded fractions (Kaiser and Berhe 2014; Schmidt et al. 1999). Furthermore, preliminary testing showed that differences in recovery were negligible at higher sonication energies (< 590 J mL^−1^) in our silty-loam textured soils. Furthermore, a higher density cut-off for our fractionation method would have likely increased recovery in the occluded fractions as was recently seen in Vormstein et al. (2020; > 1.8 gcm^-3^); but, this may have also increased mineral contamination in the LFs. Thus, evidence provided by our study implies that Ca-mediated occlusion may not be as important to the accumulation of SOC in Ca-rich humid environments as first expected.

A recent study by Yang et al. (2020) investigating mineralisation rates of incubated aggregates from similar humid, grassland, CaCO_3_-bearing or CaCO_3_-free soils (Peruvian Andes) may further support this finding. They demonstrated that mineralisation rates for incubated aggregates did not change upon their destruction, and thus, the removal of the physical separation afforded by occlusion (Yang et al. 2020). From this result, Yang et al. (2020) concluded that occlusion/physical separation was less important for the stabilisation of SOC in these humid environments, relative to mineral association. Our results would support their hypothesis and suggest that future studies investigating SOC dynamics in similar Ca-rich and humid soil environments should instead focus on the processes that increase mineral-associated SOC content.

### Ca prevalence is linked to an increased mineral-associated SOC content

It was the mineral-associated SOC pool that accounted for the majority of SOC at both sites. Since SOC content was almost twice as high at the CaCO_3_-bearing site, the mass of mineral-associated SOC was also approximately twice as high. This accumulation of mineral-associated SOC was unlikely to have arisen as a direct result of CaCO_3_ due in part to the humid conditions at the Nant Valley precluding extensive precipitation of pedogenic carbonates and the low CaCO_3_ content of the CaCO_3_-bearing profiles. Yet, we can hypothesise that CaCO_3_ likely played an indirect role in the accumulation of mineral-associated SOC at our sites, through its cascading influence on soil biogeochemistry (Rowley et al. 2020).

During its dissolution, CaCO_3_ releases Ca^2+^ into the soil solution and carbonate equilibria can buffer soil pH. In turn, this Ca source can influence the crystallinity of Fe oxides (Thompson et al. 2011). Rowley et al. (2020) indeed reported a higher incidence of poorly crystalline Fe forms (oxalate-to-dithionite extractable Fe; McKeague and Day 1966) at the studied CaCO_3_-bearing site. Both this released Ca (Rasmussen et al. 2018; Rowley et al. 2018) and the higher proportion of poorly crystalline Fe forms (Kramer and Chadwick 2018; Parfitt and Childs 1988) have well-established links to an accumulation of mineral-associated SOC through sorption processes. Yet, the direct role of poorly crystalline Fe in the sorption and stabilisation of mineral-associated SOC at the CaCO_3_-bearing site would have likely been limited by the high soil pH conditions (Sowers et al. 2018a). As pH increases, the variable surface charge of poorly crystalline Fe forms, like ferrihydrite, shifts towards negative, reducing their interaction with SOC functional groups and increasing their interaction with cations, like Ca^2+^ (Schwertmann and Fechter 1982; Sowers et al. 2018a). Following this, Ca^2+^ addition has been shown to increase the sorption of dissolved organic C by ferrihydrite at higher pH conditions, beyond its point of zero charge (Sowers et al. 2018b) in Fe-Ca-ternary complexes (Sowers et al. 2018a). This mechanism brings to light the importance of Ca^2+^ in the interactions between SOC and poorly crystalline Fe forms in soil environments with a higher pH, like the CaCO_3_-bearing site.

It is thus likely that the higher Ca content played a fundamental role in the near-doubling of mineral-associated SOC content at the CaCO_3_-bearing site, which was likely achieved through cation bridging of organo-mineral associations. Furthermore, we can speculatively hypothesise that due to the persistence of these complexes during fractionation with SPT (high Na^+^ content and cation exchange potential), these interactions are unlikely to have been outer sphere in nature and were instead, most likely inner sphere bridge complexes (Kalinichev and Kirkpatrick 2007; Rowley et al. 2018; Sutton et al. 2005).

### The complexation of SOC by calcium

We attempted to use the XPS C_1s_ and Ca_2p_ spectra (Fig. 7) to investigate the complexation of specific SOC functional groups by Ca^2+^. The complexation of SOC by Ca^2+^ has typically been thought to preferentially stabilise phenol and carboxyl functional groups (Kaiser 1998; Römkens and Dolfing 1998). The main difference between our sites in the XPS C_1s_ peak deconvolution was an increased proportion of carbonyl C moieties at the CaCO_3_-bearing site. Speculatively, this may be caused by a shift in the C_1s_ spectra upon complexation of carboxyl functional groups by Ca, as witnessed by Demri and Muster (1995; Ca[COOH]_2_ closer to 288.6 eV than 289.5 eV; Table 2). Similar shifts in the C_1s_ spectra upon complexation by different metals have indeed been previously reported in X-ray absorption spectra (De Stasio et al. 2005; Plaschke et al. 2005). However, more in-depth analyses with XPS or synchrotron-based spectroscopy (see Prietzel et al. 2020 for more details) would be required to investigate this hypothesis as the Ca_2p_ spectra could not provide supporting evidence.

There were two peaks present in the Ca_2p_ spectra at the CaCO_3_-bearing site, but only one weaker peak present at the CaCO_3_-free site (Fig. 7). Unfortunately, this peak could not be accurately identified by XPS as there is only a small range of chemical shifts in the Ca_2p_ spectra (< 1 eV; Moulder and Chastain 1992) and XPS data on Ca-rich soil samples has not been widely reported in the literature (Boiteau et al. 2020; Demri and Muster 1995). We can clearly state that the peaks in our Ca_2p_ region were not related to CaCO_3_, since it had been quantitatively removed by the HCl fumigation. Boiteau et al. (2020) recently attributed similar peaks to Ca–O–C bonds and Ca-plagioclase. Speculatively, the Ca_2p3/2_ peak at the CaCO_3_-bearing site is also likely linked to similar Ca–O–C bonds, inherent Ca-bound within particulate OM, and potentially some Ca-plagioclase (Boiteau et al. 2020; Rowley et al.^2020^). More advanced spectroscopic methods are now required to confirm this hypothesis and identify whether inner sphere complexes mediated by Ca^2+^ are indeed responsible for the increased mineral-associated SOC content of these soils.

### Organic matter quality

#### Bulk soil differences

Bulk *δ*^13^C values were lower at the CaCO_3_-bearing site relative to the CaCO_3_-free site, even though vegetation *δ*^13^C values were largely invariant (Fig. 2). Furthermore, while the CaCO_3_-bearing site had a lower proportion of A5 contribution to the S2 thermogram (most-thermally stable SOC), it also had lower *I Index* scores in the B horizons (less thermally stable; Fig. 3). This suggests that there is an accumulation of SOC with a more moderate and homogeneous thermal signature at this site. In contrast, OM at the CaCO_3_-free site had a relatively higher proportion of compounds with both low and high thermostability. These results contrasted previous results for the Alps, which demonstrated that CaCO_3_-bearing soils typically had lower *R index* scores. This contrast could potentially be explained by the scale of our different studies; where, Rock-Eval® analyses may have struggled to identify the differences between SOC at our highly similar sites, relative to the geochemically diverse dataset of Matteodo et al. (2018; Suppl. Fig. S6) or pedoclimatically diverse dataset of Sebag et al. (2016).

Both bulk thermostability (Sebag et al. 2016) and *δ*^13^C values (Boström et al. 2007) are commonly assumed to increase during decomposition processes in soils; yet these signatures can also be influenced by the composition of specific organic compounds or their preferential stabilisation by the mineral phase, due to polarity or stearic constraints. Therefore, it is reasonable to suggest that the trends we observe are the result of both continued decomposition processes and the preferential stabilisation of specific organic compounds at each site, driven by fundamental differences in their mineralogy and bulk geochemistry. Based on our data, we can hypothesise that active decomposition and mineralisation processes were operant at the CaCO_3_-free site; which meant that OM composition was dominated either by relatively fresh plant material that had not yet fully entered the decomposition continuum or highly decomposed residues from active decomposition and mineralisation processes. This would result in a thermal signature with a relatively increased presence of thermally labile compounds (high *I index*), but also thermally stable compounds representing advanced decomposition residues (A5; Malou et al. 2020). This proposition is also consistent with the observation that the bulk SOC contents were lower and the *δ*^13^C values were higher at the CaCO_3_-free site. On the other hand, at the CaCO_3_-bearing site, the intensity of decomposition processes might have been inhibited by Ca^2+^; which may have mediated a preferential stabilisation of organic compounds with *δ*^13^C values that are indicative of slight oxidative transformation and a moderate thermostability.

Differences in the microbial community composition, activity, or abundance were not measured at the Nant Valley. Microorganism communities have different biogeochemical mechanisms for the oxidative transformation of SOC or the utilisation of Ca; which in turn, drive variations in their C use efficiency, the partitioning of C between microbial biomass and respiration (Bradford and Crowther 2013), or the stability of Ca minerals or complexes (Gadd 2010). Soil pH, which increased from the CaCO_3_-free to the CaCO_3_-bearing site, is known to act as a ‘master variable’ in soils, and an increase in pH is linked to shifts in microbial community composition (decreasing fungal to bacterial ratio) and/or functioning (Bahram et al. 2018; Blagodatskaya and Anderson 1999; Rousk et al. 2010; Rousk et al. 2009). Soares and Rousk (2019) recently demonstrated that C use efficiency had an exponential negative relationship with the fungal to bacterial ratio. It can be hypothesised that an increase in pH at our CaCO_3_-bearing sites may have been linked to a decrease in the fungal-to-bacterial ratio (Bahram et al. 2018; Blagodatskaya and Anderson 1999; Rousk et al. 2010; Rousk et al. 2009). Hypothetically, this shift may have increased carbon use efficiency at the CaCO_3_-bearing site, which would have caused an accumulation of SOC as microbial biomass and necromass (Bahram et al. 2018; Rousk et al. 2010; Rousk et al. 2009). Future studies should investigate how variations in Ca content can influence microbial communities, their carbon use efficiency, and its influence on SOC accumulation or quality.

#### Similar *δ*^13^C values in the HF and LF

At the CaCO_3_-free site, *δ*^13^C values of the LFs were lower than the HF (Fig. 5), displaying a typical shift from less to more oxidatively transformed C moving from particulate OM to mineral-associated SOC, respectively (Poeplau et al. 2017; Schrumpf et al. 2013). This contrasted with the CaCO_3_-bearing site where *δ*^13^C values were similar between the LFs and HF (Fig. 5). As described in "Materials and methods", this is unlikely to have arisen due to the precipitation of Ca metatungstate. Instead, as our sites have developed under similar soil forming conditions, these differences can likely be linked to the variation in Ca content.

In a recent study, Martí-Roura et al. (2019) used a size fractionation method on CaCO_3_-free and CaCO_3_-bearing Mediterranean soils. Similar to our findings with DF, they demonstrated that CaCO_3_-bearing soils displayed a smaller shift in *δ*^13^C values between the coarse and fine size fractions (Martí-Roura et al. 2019). It therefore seems that the similarity between different fractions at CaCO_3_-bearing sites could be independent of fractionation scheme and is instead related to a natural process. The *δ*^13^C values of the fractions insinuated that the LFs were more oxidatively transformed at the CaCO_3_-bearing site, but the HF were less oxidatively transformed (Fig. 5). This hypothesis is in accordance with the bulk Rock-Eval^®^ measurements, in which the CaCO_3_-bearing site contained more moderately thermostable OM and the CaCO_3_-free site contained both less- and the most-thermally stable OM. These observations provide further support for the hypothesis that there was a preferential stabilisation of SOC with lower *δ*^13^C values and moderate thermostability in the mineral or finer-size fractions of soils with an increased Ca prevalence.

### The decomposition continuum in Ca-rich soils

We can thus speculate that together, the bulk Rock-Eval^®^ signatures, similarity between LFs and HF *δ*^13^C values (Fig. 5), and increased HF SOC content at the CaCO_3_-bearing site may all support the *Decomposition Continuum* model in Ca-rich soils (Kleber and Lehmann 2019; Lehmann and Kleber 2015). According to this model, oxidative transformation by microorganisms increases the proportion of negatively-charged functional groups in SOC, which subsequently increases its reactivity towards minerals or cations (Lehmann and Kleber 2015). During the complexation of these functional groups through cation bridging processes, Ca^2+^ could be preferentially preserving SOC that had already passed a certain level of oxidative transformation at the CaCO_3_-bearing site. Once this level of oxidative transformation was achieved, SOC functional groups could be complexed by Ca^2+^. Thereafter, complexation processes with Ca^2+^ seem to inhibit the complete mineralisation of SOC, causing an accumulation of slightly oxidatively transformed SOC in the mineral-associated fraction of soils with a larger Ca prevalence (CaCO_3_-bearing). More investigation is now required to confirm this hypothesis and further probe the complexation of SOC by Ca^2+^ in different soil environments, increasing our understanding of the mechanisms and kinetics of these interactions.

## Conclusions

To isolate the complex role of Ca in SOC accumulation, we performed a fractionation study on soils which had formed under similar conditions but were either CaCO_3_-bearing or CaCO_3_-free. Bulk SOC was twice as high at the CaCO_3_-bearing profiles, which also had lower *δ*^13^C values and a moderate thermostability. Occluded SOC pools were larger at the CaCO_3_-bearing site but were of little overall significance to bulk SOC dynamics at either of our sites. It was instead the HF that accounted for most of the total organic C. The HF thus contained almost twice as much SOC at the CaCO_3_-bearing site, establishing that soils with an increased Ca prevalence had a two-fold increase in mineral-associated SOC storage, relative to similar soils with less Ca.

The *δ*^13^C values of the density fractions from samples at the CaCO_3_-free site displayed a typical increase from less to more processed OM between the LFs and HF, respectively. This contrasted with the CaCO_3_-bearing site, which had similar *δ*^13^C values in the HF and LFs. Both these similarities between the LFs and HF, and the accumulation of mineral-associated SOC at the CaCO_3_-bearing site were most likely driven by the preferential complexation of SOC in organo-mineral associations mediated by Ca. Through increasing the relative proportion of negatively-charged functional groups, decomposition may actually increase the propensity of SOC to be stabilised through complexation with Ca; thereby, preventing its complete mineralisation and causing it to accumulate in the mineral-associated fraction of soils with an increased Ca prevalence (CaCO_3_-bearing). Future investigations should now investigate Ca-mediated complexation processes, how they may be applied to increase global SOC stocks (Minasny et al. 2017), and their potential interactions with management practices that aim to sequester C inorganically (Beerling et al. 2020).

## Supplementary Information

Below is the link to the electronic supplementary material.

Supplementary material 1 (DOCX 12677kb)

## Data Availability

We have been fully transparent with our data and materials, including four separate tables in the supplementary information. There is no code to be made available, but we have fully detailed our techniques in "Materials and methods".

## Abbreviations

AGB: Above-ground biomass
BGB: Below-ground biomass
*δ*^13^C: C stable isotope ratio in per mil (‰) relative to the Vienna Pee Dee Belemnite standard.
DF: Density fractionation
f-LF: Free-light fractions
HF: Heavy fractions/mineral-associated fractions
LF: Light fractions (f-LF, o-LF_10_, o-LF_200_)
o-LF_10_: Occluded light fractions separated at 10 J mL^−^^1^
o-LF_200_: Occluded light fractions separated at 200 J mL^−^^1^
OM: Organic matter
SE: Standard error of the mean
SOC: Soil organic carbon
SPT: Sodium polytungstate
XPS: X-ray photoelectron spectroscopy
