# Supplementary material for: Evidence linking calcium to increased organo-mineral association in soils
Source: Biogeochemistry. 2021 Apr 4;153(3):223–41. doi: 10.1007/s10533-021-00779-7 (PMC8550578; doi:10.1007/s10533-021-00779-7)
Supplement: Supplementary file 1 — Supplementary material 1 (DOCX 12677kb) [file 10533_2021_779_MOESM1_ESM.docx]

# Supplementary Figures.


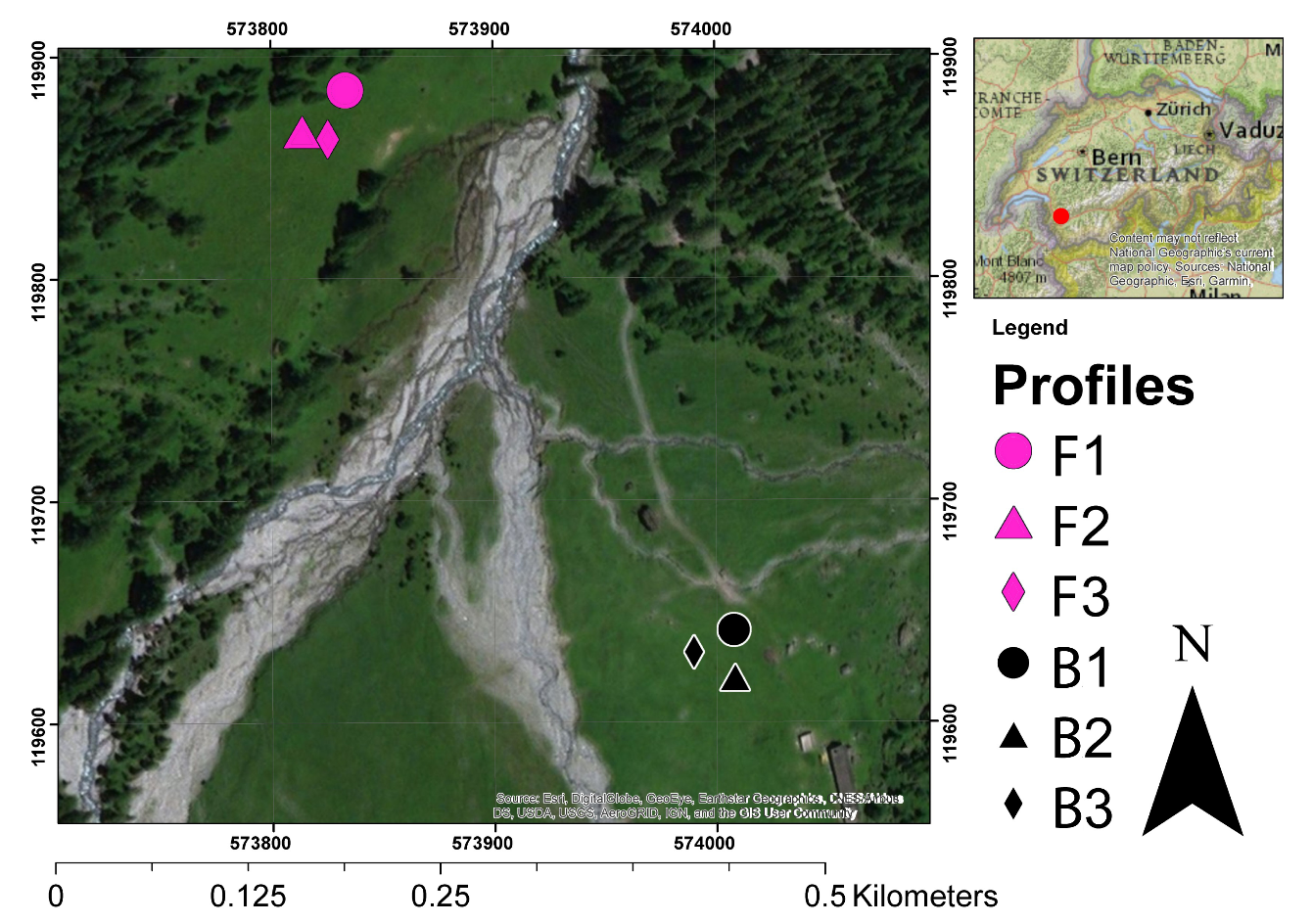


**Suppl. Fig. 1.** Profile locations at the Nant Valley rangeland, Vaud Alps, Switzerland (ESRI, 2019). Coordinates are in CH1903 LV03. **Calcium carbonate-free** profiles are labelled with an **F (F1, F2, F3)** and **CaCO_3_-bearing** profiles are labelled with a **B (B1, B2, B3).**


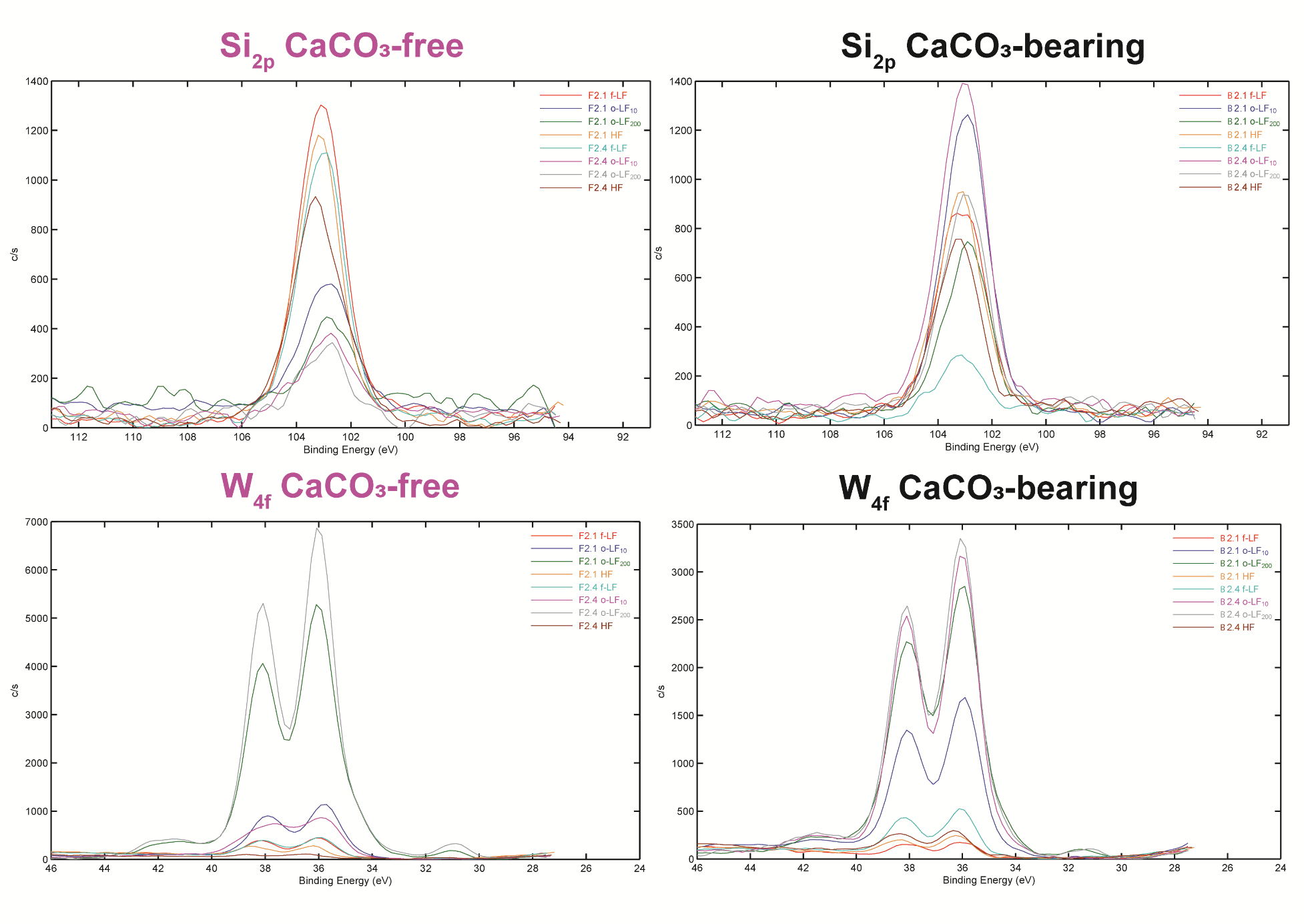


Suppl. Fig. 2. Detailed XPS spectra in the Si_2p_ and W_4f_ binding energy range of all density fractions from the CaCO_3_-bearing (B2.1 & B2.4) and CaCO_3_-free (F2.1 & F2.4) sample subset. Significant precipitation of Ca-metatungstate would shift the tungstate peak from 35.5-36 eV towards 35 eV (Moulder and Chastain, 1992).


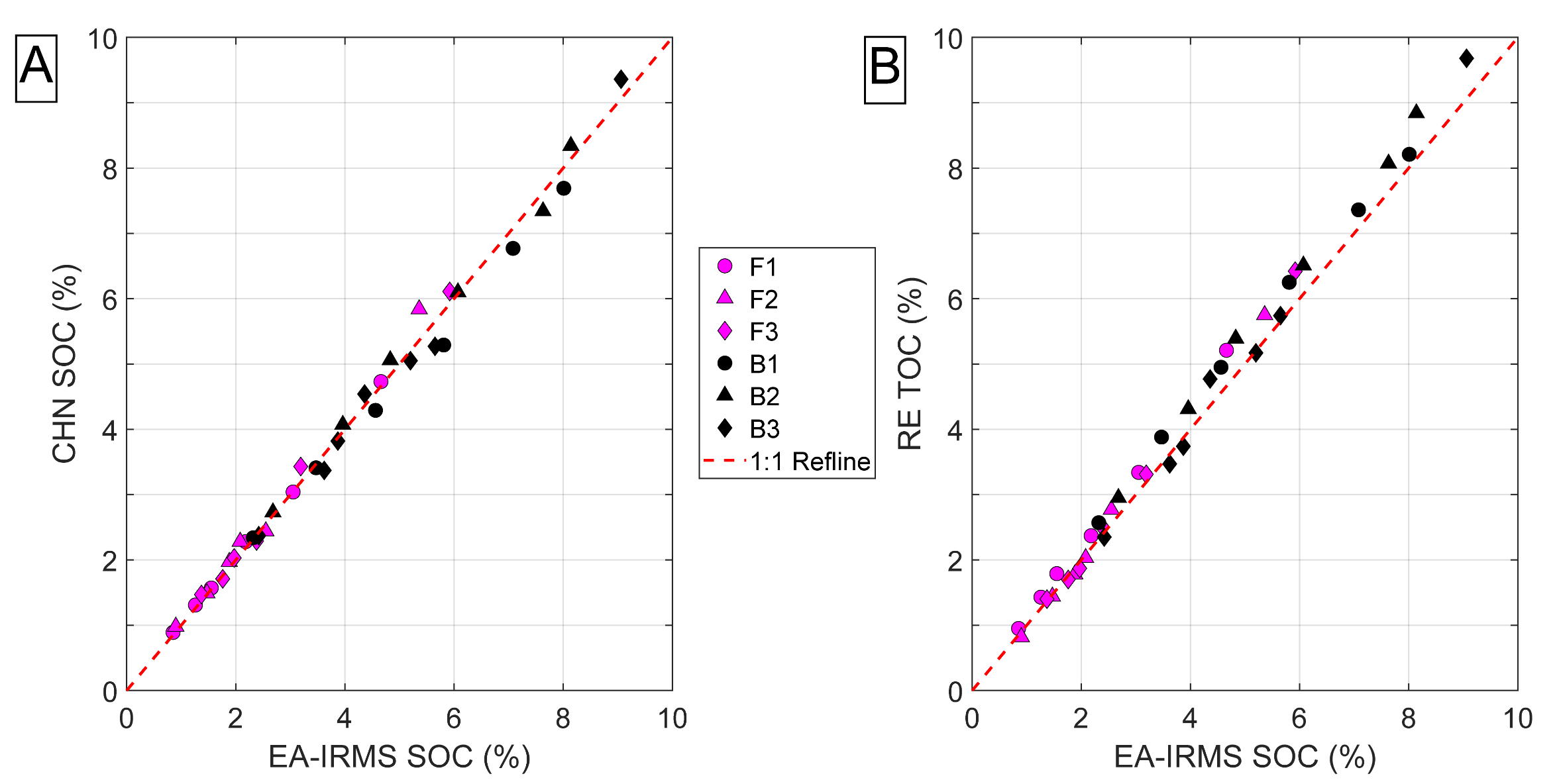


Suppl. Fig. 3 A & B. Comparison of bulk soil organic carbon (SOC) content measured with different instruments on samples from the **CaCO_3_-free (F1, F2, F3)** and **CaCO_3_-bearing (B1, B2, B3)** profiles. **A)** Direct comparison of the Elemental Analyser measurements of SOC from Rowley et al. (2020; CHN) with the Elemental Analyser Isotope-ratio Mass Spectrometer (EA-IRMS) measurements from this study. **B)** Comparison of Rock-Eval (RE) measurements of total organic carbon (RE TOC) with the EA-IRMS SOC measurements. Relative to measurements with the Elemental Analysers from this study and Rowley et al. (2020), the Rock-Eval systematically overestimated total organic carbon (TOC) content while underestimating mineral C content of the CaCO_3_-bearing samples. This was most likely caused by the erroneous attribution of minor amounts of inorganic C to TOC estimates. However, the overall offset between the measurements was small (< 0.7 %).


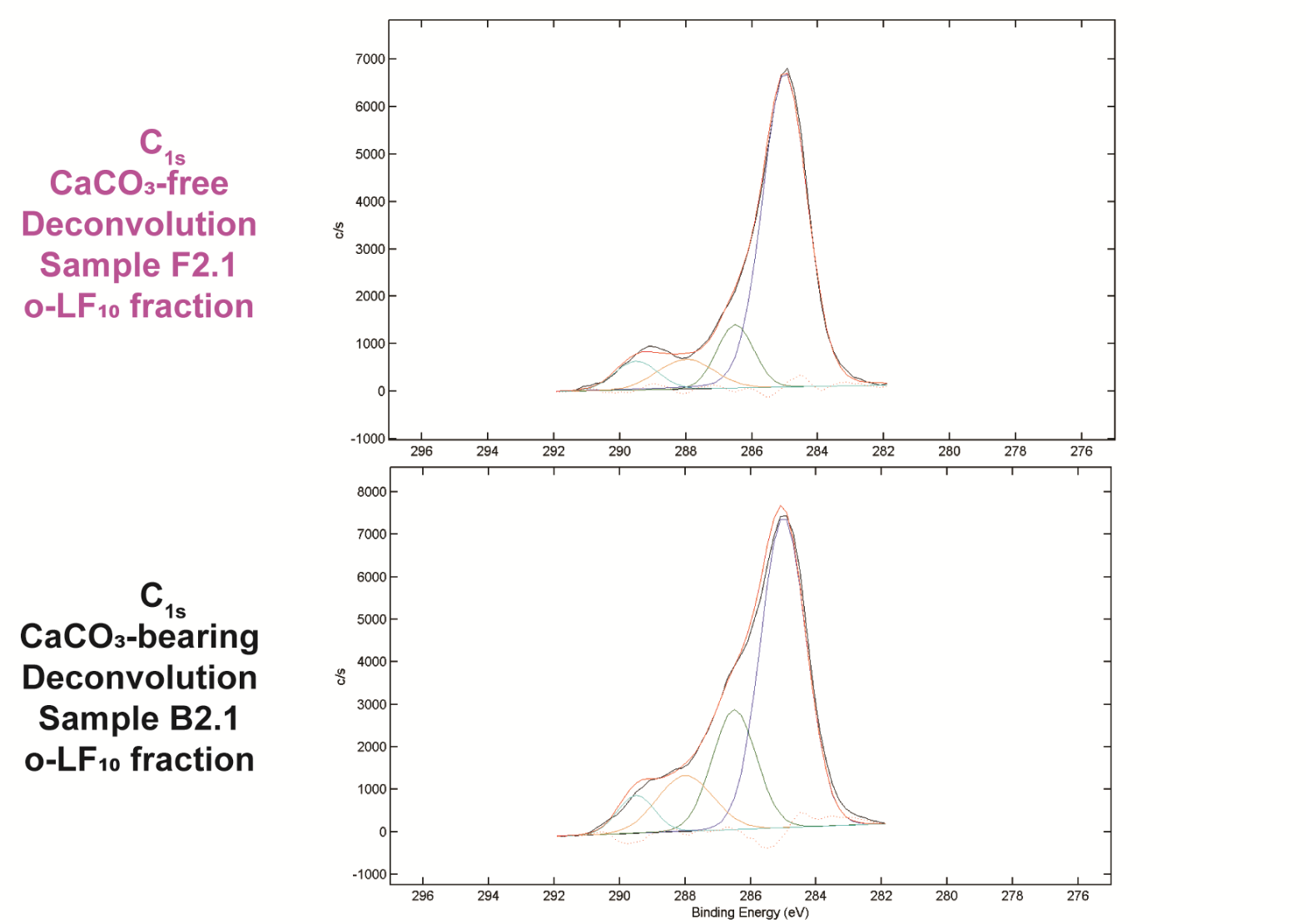


Suppl. Fig. 4. Example of the deconvolution of the X-ray photoelectron attained C_1s_ spectra. Spectra were deconvoluted into four different sub-peaks, representing different C bonding environments (from left to right): carboxylate (289.5 eV), carbonyl (288 eV), alcoholic / phenolic (286.5 eV), aromatic / aliphatic (285 eV) moieties.


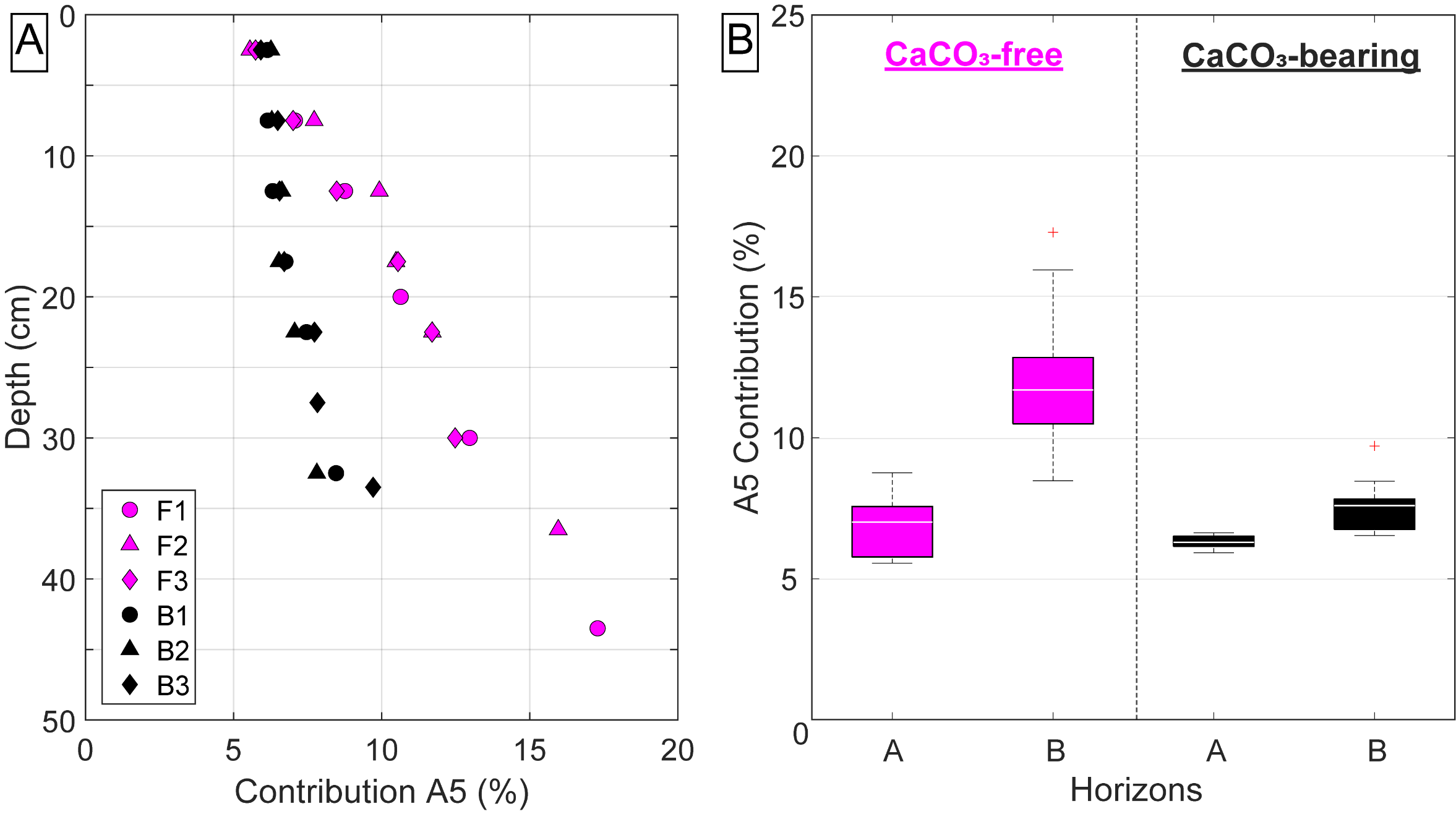


Suppl. Fig. 5 A) The A5 contribution to the S2 thermogram deconvolution at the CaCO_3_-free (F1, F2, F3) and CaCO_3_-bearing (B1, B2, B3) profiles. B) Direct comparison of the A5 contributions in A and B horizons of the two sites. Bottom and top edges of the boxes in the box plot represent the 25^th^ and 75^th^ percentiles, the middle bars represent the median. Whiskers represent the range of data points not considered as outliers, while ‘+’ represent values outside of the maximum potential whisker value, corresponding to ±0.4 standard error of the mean (outliers).


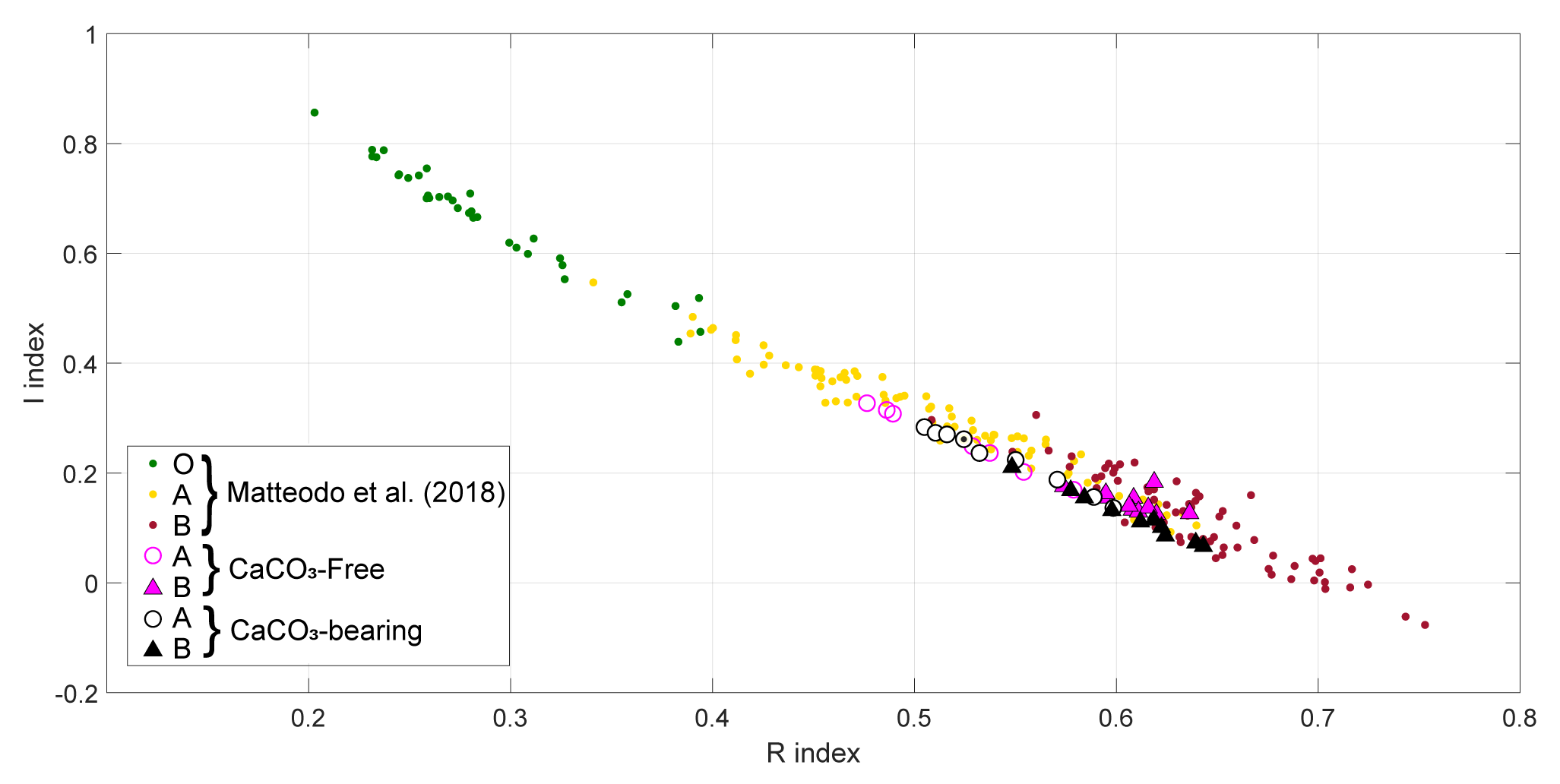


Suppl. Fig. 6. Rock-Eval I and R thermal stability indices from this study and other alpine soils in Switzerland from a geochemically diverse dataset (Matteodo et al., 2018).


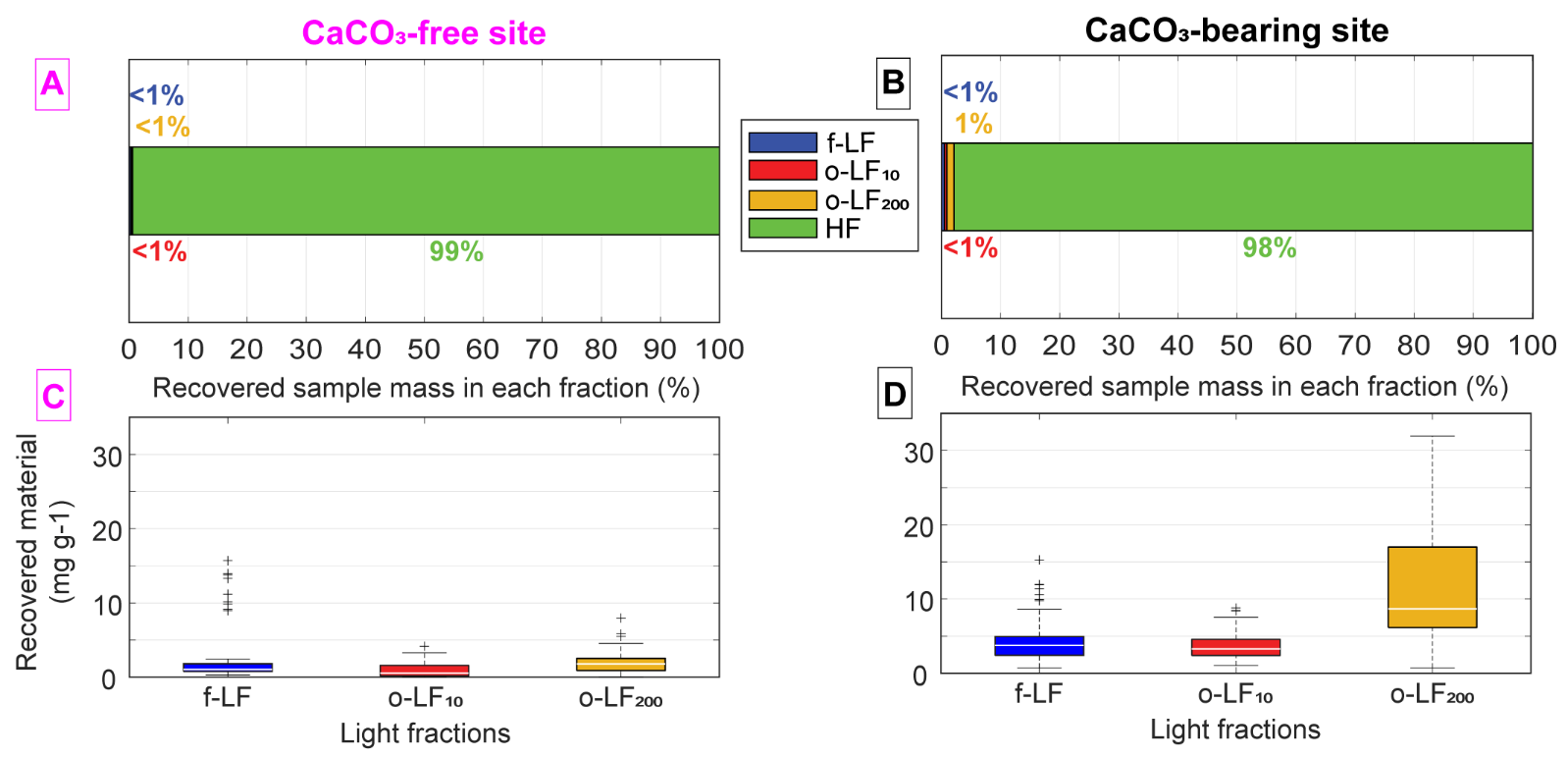


Suppl. Fig. 7. **A & B)** Average sample mass recovered in the fractions (free-light, occluded light fractions separated at 10 J mL^-1^ and 200 J mL^-1^, and heavy fractions; f-LF, o-LF_10_, o-LF_200_, HF, respectively) from the **(A)** **CaCO_3_-free** and **(B)** **CaCO_3_-bearing** site. **C & D)** Average sample mass recovered in the light fractions per gram of oven dried (105°C) fractionated soil (mg g^-1^) from the **(C) CaCO_3_-free** and **(D)** **CaCO_3_-bearing** site. Bottom and top edges of the boxes in the box plot represent the 25^th^ and 75^th^ percentiles, the middle bars represent the median. Whiskers represent the range of data points not considered as outliers, while ‘+’ represent values outside of the maximum potential whisker value, corresponding to ±0.4 standard error of the mean (outliers).


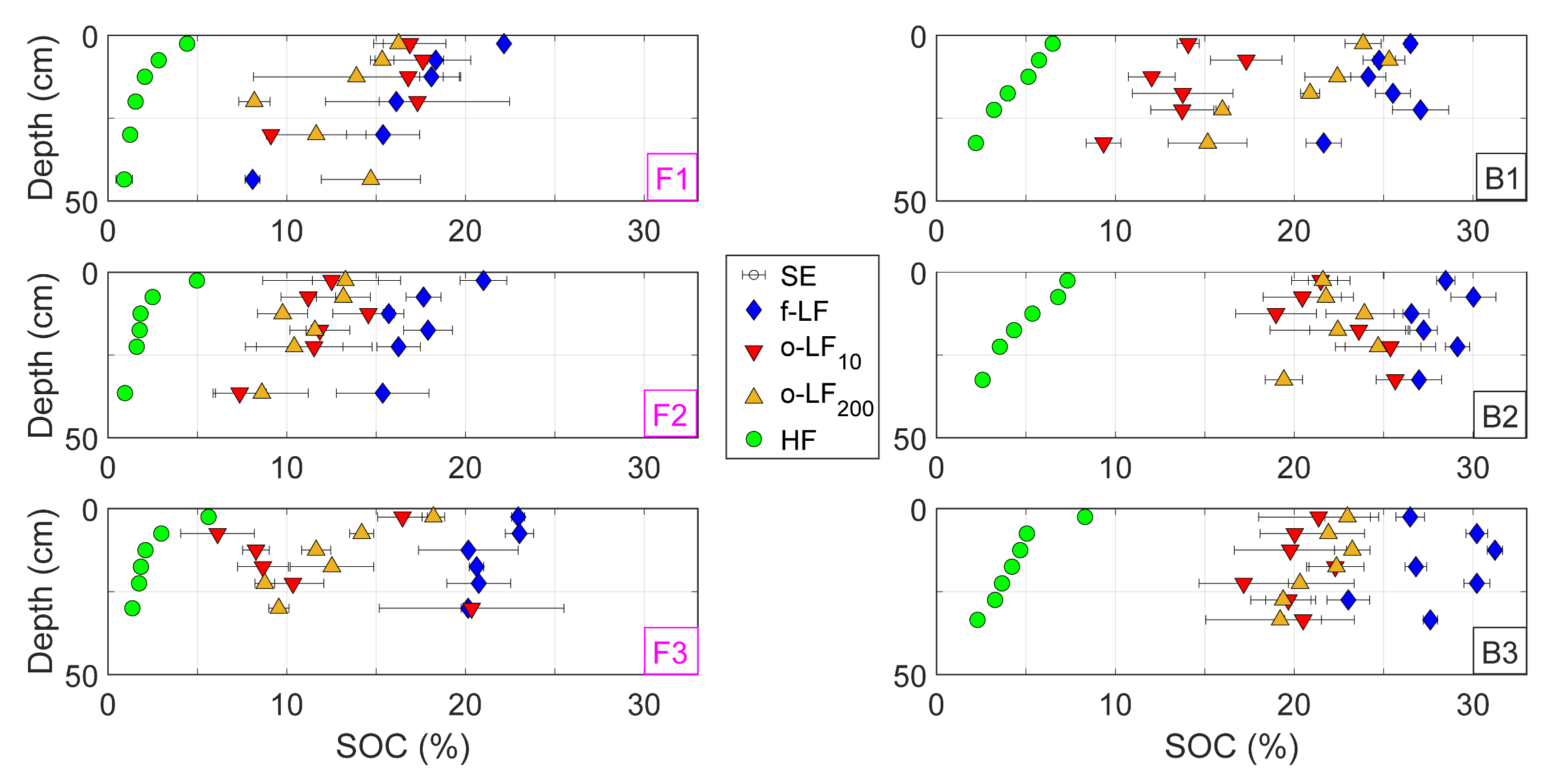


Suppl. Fig. 8. Mean soil organic carbon (SOC) content of the free-light fraction (f-LF), occluded fractions separated at 10 J mL^-1^ (o-LF_10_) and 200 J mL^-1^ (o-LF_200_) and heavy fraction (HF). The **CaCO_3_-free (F1, F2, F3)** profiles are on the left and the **CaCO_3_-bearing (B1, B2, B3)** profiles are on the right. Error bars represent the standard error of the mean (SE) between triplicate measurements. The amount of material recovered in o-LF_10_ of F1.6 was insufficient for analysis.


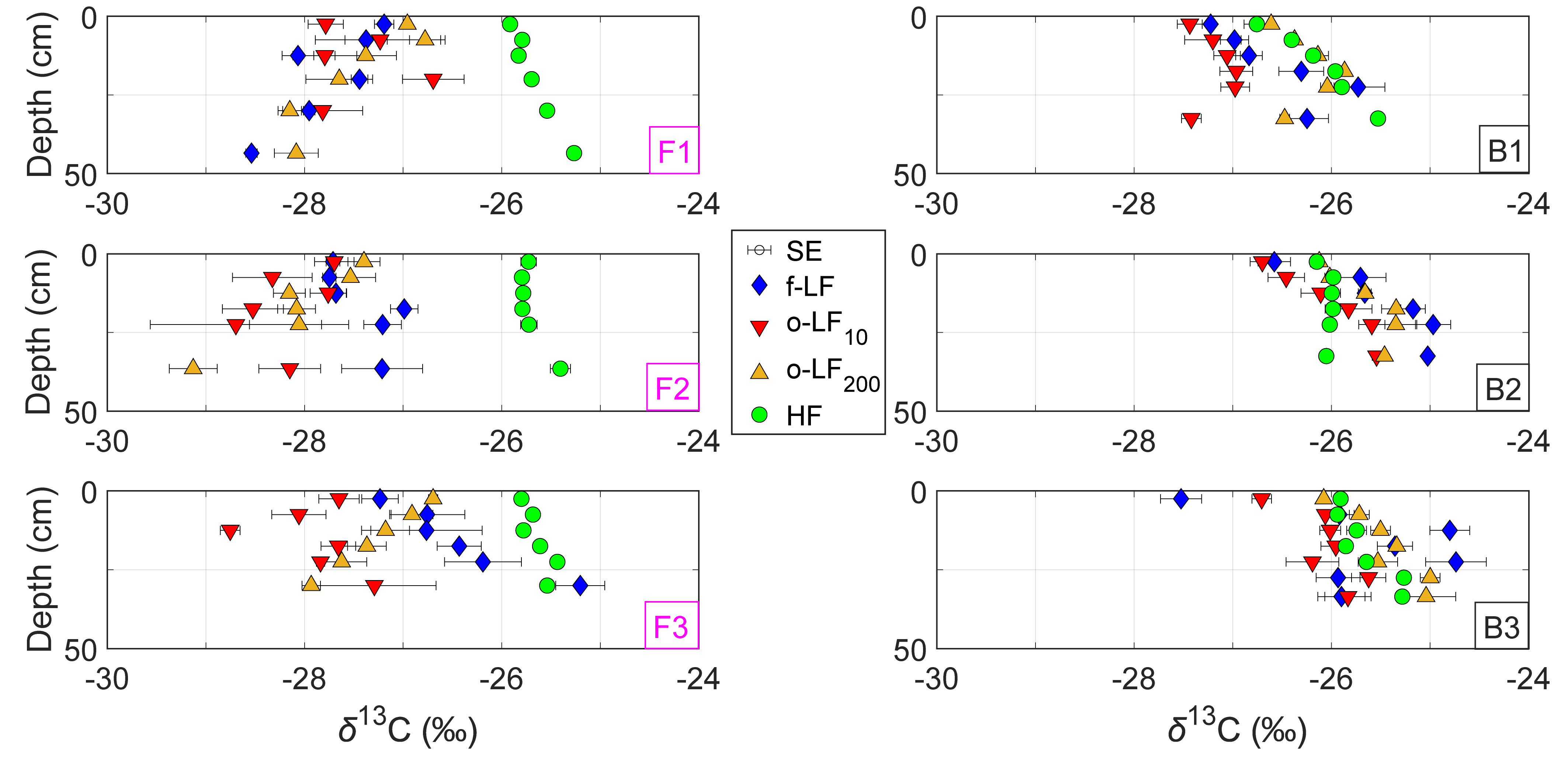


Suppl. Fig. 9. Mean carbon stable isotope compositions (*δ*^13^C values) of the free-light fraction (f-LF), occluded fractions separated at 10 J mL^-1^(o-LF_10_) and 200 J mL^-1^(o-LF_200_) and heavy fraction (HF). The **CaCO_3_-free (F1, F2, F3)** profiles are on the left and the **CaCO_3_-bearing (B1, B2, B3)** profiles are on the right. Error bars represent the standard error of the mean (SE) between triplicate measurements. The amount of material recovered in the o-LF_10_ of F1.6 was insufficient for analysis.


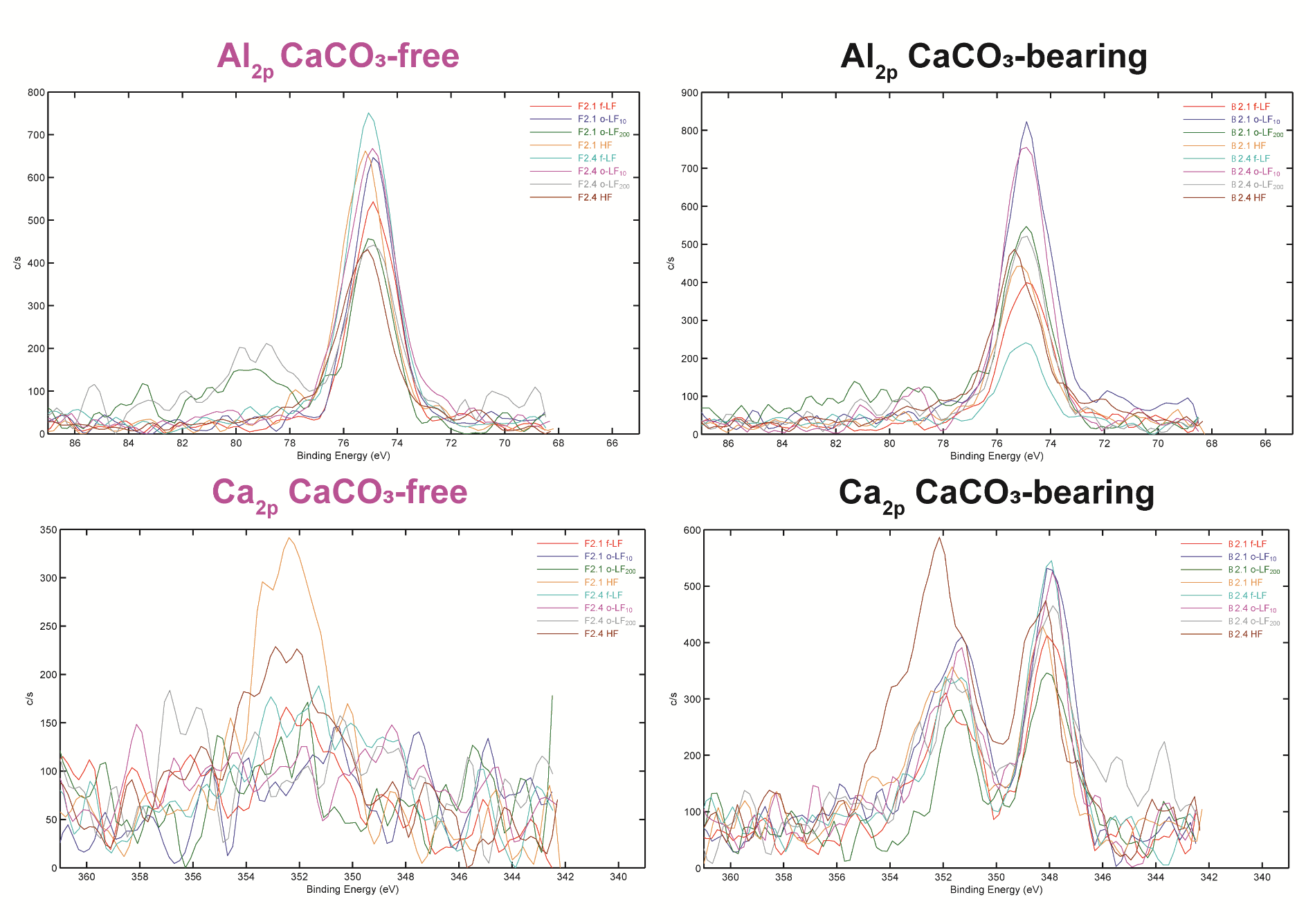


Suppl. fig. 10. Detailed XPS spectra in the Al_2p_ and Ca_2p_ binding energy ranges of all density fractions from the CaCO_3_-bearing (B2.1 & B2.4) and CaCO_3_-free (F2.1 & F2.4) sample subset. The Ca_2p3/2_ peak is difficult to distinguish due to the lack of a chemical shift in the Ca_2p_ region, lack of pre-existing XPS data on Ca-C-O interactions and location of the peak. The Ca_2p3/2_ peak could be attributed to CaCl_2_ or Ca-O type bonds (not CaCO_3_ as it had been removed with an HCl fumigation).


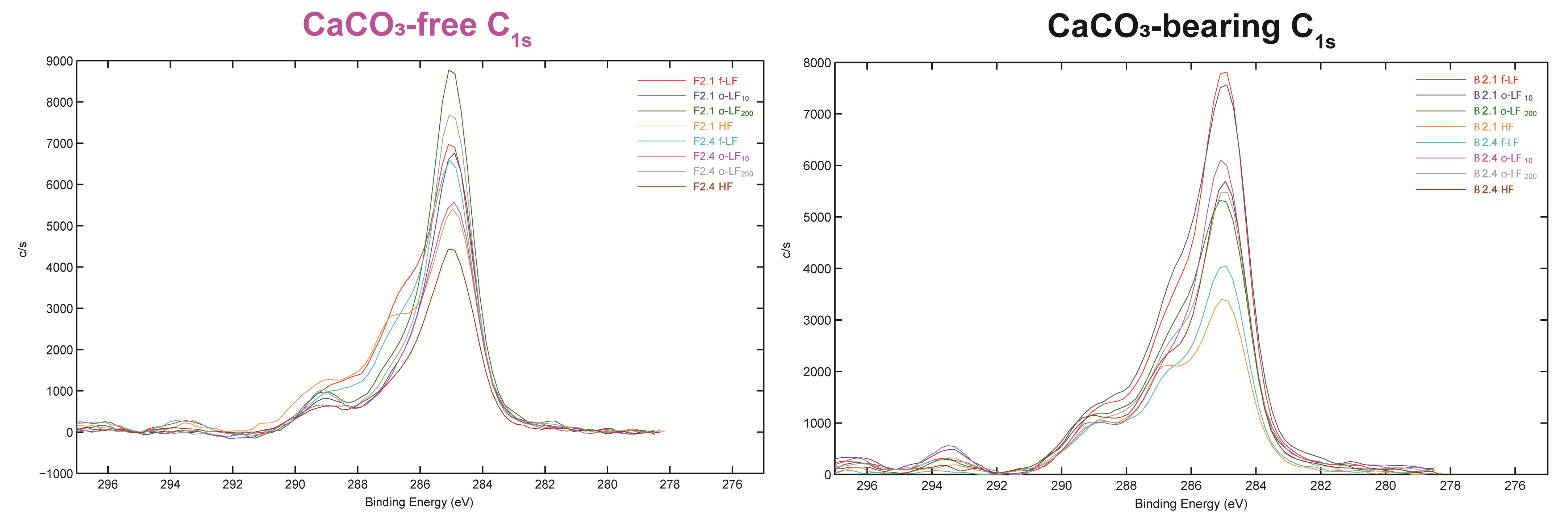


Suppl. Fig. 11. Detailed XPS spectra in the C_1s_ binding energy range of all density fractions from the CaCO_3_-bearing (B2.1 & B2.4) and CaCO_3_-free (F2.1 & F2.4) sample subset.


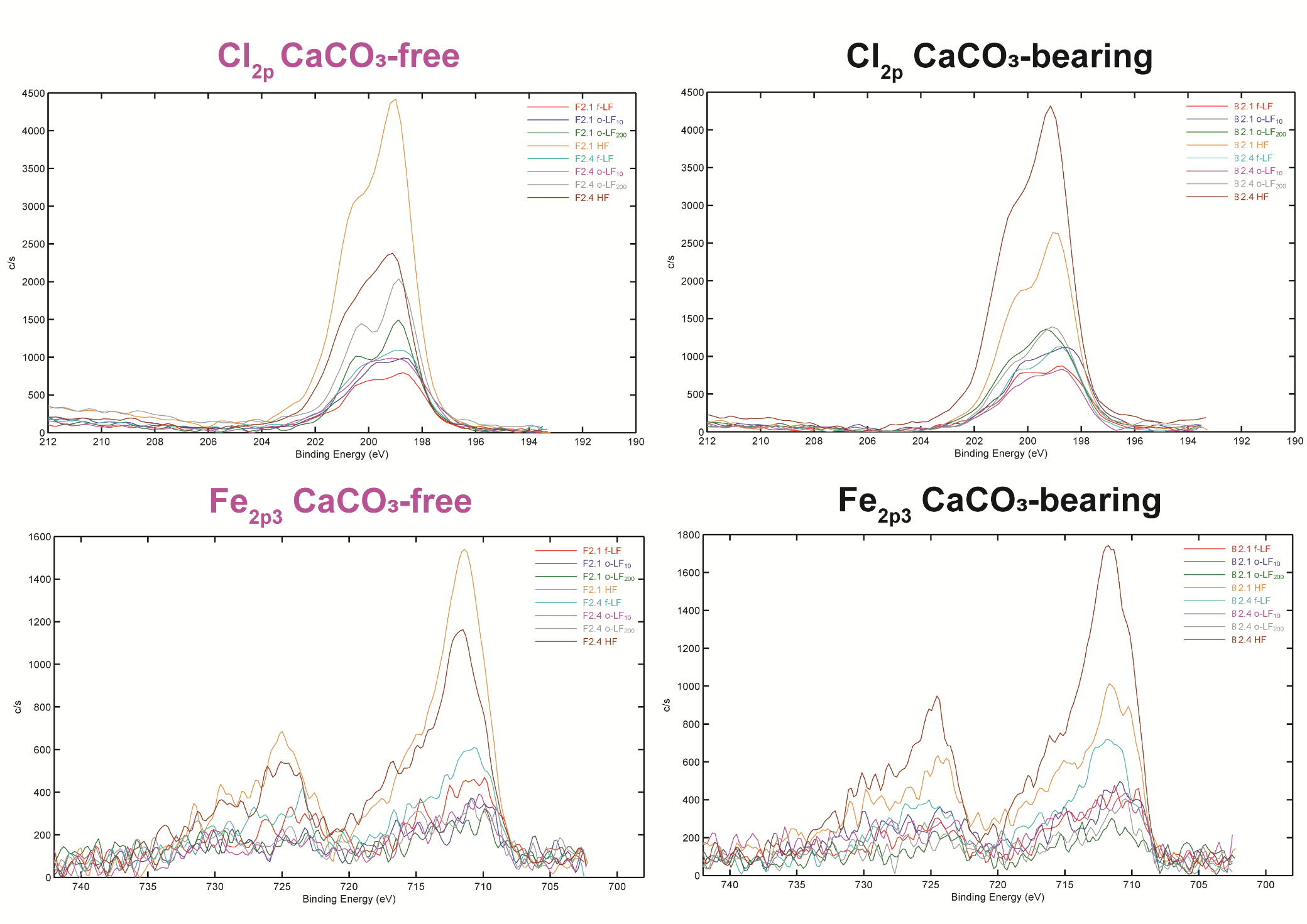


Suppl. Fig. 12. Detailed XPS spectra in the Cl_2p_ and Fe_2F3_ binding energy range of all density fractions from the CaCO_3_-bearing (B2.1 & B2.4) and CaCO_3_-free (F2.1 & F2.4) sample subset.


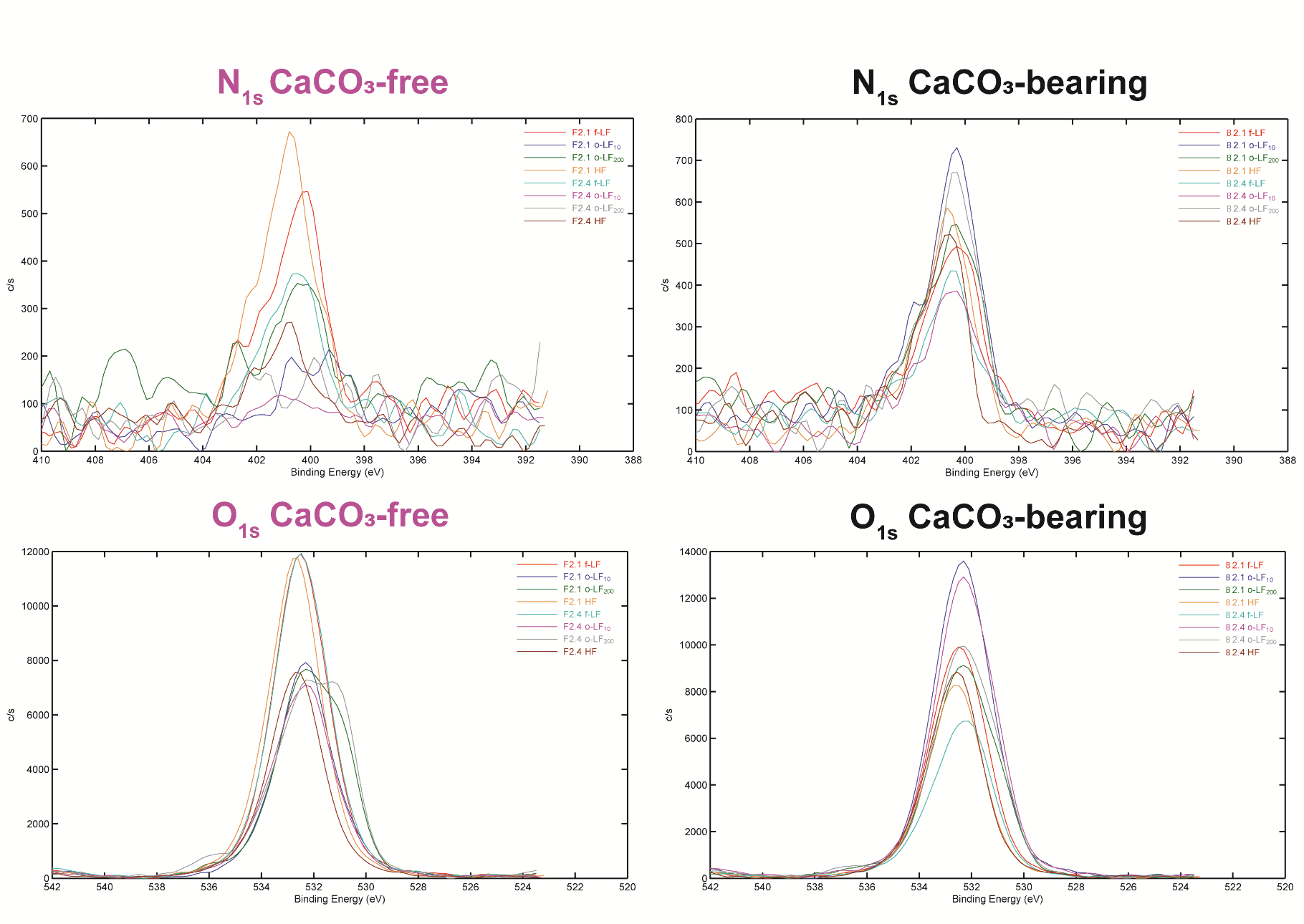


Suppl. Fig. 13. Detailed XPS spectra in the N_1s_ and O_1s_ binding energy range of all density fractions from the CaCO_3_-bearing (B2.1 & B2.4) and CaCO_3_-free (F2.1 & F2.4) sample subset. There is a slight shift towards more protonated N_1s_ forms at the CaCO_3_-free site, which has a more acidic pH (Jones and Singh, 2014).

# Supplementary tables

Suppl. Table 1. Mass recovery, soil organic carbon (SOC) content and mass of SOC in each density and sonication separated from the **CaCO_3_-free (F1, F2, F3)** and **CaCO_3_-bearing** **(B1, B2, B3)** profiles. There was insufficient material recovered in F1.6 o-LF_10_ for analysis. All figures are mean values of the triplicated fractionation plus or minus the standard error of the mean.

| **Sample** | **Fraction mass** | | | | | **SOC content** | | | | | **Mass of SOC** | | | | |
| --- | --- | --- | --- | --- | --- | --- | --- | --- | --- | --- | --- | --- | --- | --- | --- |
|  | **f-LF** | **o-LF_10_** | **o-LF_200_** | **HF** | **Recovery**  **rate** | **f-LF** | **o-LF_10_** | **o-LF_200_** | **HF** | **f-LF** | | **o-LF_10_** | | **o-LF_200_** | **HF** |
|  | **mg g^-1^** | **mg g^-1^** | **mg g^-1^** | **mg g^-1^** | **%** | **%** | **%** | **%** | **%** | **mg C g^-1^** | | | **mg C g^-1^** | **mg C g^-1^** | **mg C g^-1^** |
| **F1.1** | 9.3±0.2 | 1.2±0.2 | 3.4±0.4 | 977.4±1.2 | 99.1±0.2 | 22.2±0.1 | 16.9±2.0 | 16.3±0.9 | 4.4±0.0 | 2.1±0.0 | | | 0.2±0.0 | 0.5±0.0 | 43.2±0.3 |
| **F1.2** | 1.8±0.2 | 0.7±0.3 | 2.0±0.1 | 990.1±0.7 | 99.5±0.1 | 18.3±0.4 | 17.6±2.7 | 15.3±0.7 | 2.8±0.0 | 0.3±0.0 | | | 0.1±0.0 | 0.3±0.0 | 28.1±0.1 |
| **F1.3** | 1.2±0.0 | 0.7±0.4 | 1.7±0.7 | 993.9±1.1 | 99.8±0.2 | 18.1±0.7 | 16.8±2.9 | 13.9±5.8 | 2.1±0.0 | 0.2±0.0 | | | 0.1±0.0 | 0.1±0.0 | 20.5±0.3 |
| **F1.4** | 0.8±0.0 | 0.2±0.1 | 1.3±0.1 | 992.5±0.8 | 99.5±0.1 | 16.1±1.0 | 17.3±5.1 | 8.2±0.9 | 1.5±0.0 | 0.1±0.0 | | | 0.0±0.0 | 0.1±0.0 | 15.3±0.0 |
| **F1.5** | 0.7±0.1 | 0.2±0.0 | 0.6±0.2 | 996.8±0.8 | 99.8±0.1 | 15.4±2.0 | 9.1±0.2 | 11.6±2.8 | 1.2±0.0 | 0.1±0.0 | | | 0.0±0.0 | 0.1±0.0 | 12.3±0.1 |
| **F1.6** | 0.6±0.0 | 0 | 0.1±0.0 | 988.7±2.6 | 98.9±0.3 | 8.1±0.4 | -- | 14.7±2.8 | 0.9±0.0 | 0.0±0.0 | | | 0 | 0.0±0.0 | 8.8±0.1 |
| **F2.1** | 11.7±0.9 | 2.4±0.8 | 3.7±0.5 | 981.0±1.5 | 99.9±0.1 | 21.0±1.3 | 12.5±3.9 | 13.3±1.8 | 5.0±0.1 | 2.4±0.1 | | | 0.2±0.0 | 0.5±0.0 | 48.8±0.6 |
| **F2.2** | 1.5±0.2 | 1.5±0.4 | 1.9±0.6 | 998.2±0.4 | 100.3±0.1 | 17.7±1.0 | 11.2±1.5 | 13.2±1.5 | 2.5±0.0 | 0.3±0.0 | | | 0.1±0.0 | 0.2±0.1 | 24.7±0.3 |
| **F2.3** | 1.2±0.0 | 0.4±0.1 | 2.5±0.6 | 1000.2±0.7 | 100.4±0.1 | 15.7±0.3 | 14.6±2.0 | 9.8±1.4 | 1.8±0.2 | 0.2±0.0 | | | 0.1±0.0 | 0.2±0.0 | 18.2±1.7 |
| **F2.4** | 1.0±0.2 | 0.8±0.3 | 1.8±0.1 | 999.5±0.9 | 100.3±0.1 | 17.9±1.4 | 11.9±1.7 | 11.6±0.5 | 1.8±0.0 | 0.2±0.0 | | | 0.1±0.0 | 0.2±0.0 | 17.7±0.1 |
| **F2.5** | 0.8±0.1 | 0.4±0.2 | 1.5±0.6 | 999.5±0.7 | 100.2±0.1 | 16.3±1.2 | 11.5±3.2 | 10.4±2.7 | 1.6±0.2 | 0.1±0.0 | | | 0.0±0.0 | 0.1±0.0 | 16.0±1.7 |
| **F2.6** | 0.5±0.1 | 0.3±0.2 | 1.1±0.4 | 994.7±0.6 | 99.7±0.1 | 15.4±2.6 | 7.4±1.5 | 8.6±2.6 | 0.9±0.0 | 0.1±0.0 | | | 0.0±0.0 | 0.1±0.0 | 9.3±0.0 |
| **F3.1** | 14.3±0.6 | 2.8±0.2 | 6.4±0.6 | 968.8±1.3 | 99.2±0.2 | 23.0±0.4 | 16.5±1.4 | 18.2±0.6 | 5.6±0.0 | 3.3±0.1 | | | 0.5±0.0 | 1.2±0.1 | 54.4±0.1 |
| **F3.2** | 2.1±0.2 | 1.5±0.2 | 2.1±0.6 | 991.5±1.3 | 99.7±0.2 | 23.0±0.8 | 6.1±2.1 | 14.2±0.7 | 3.0±0.0 | 0.5±0.0 | | | 0.1±0.0 | 0.3±0.1 | 29.5±0.1 |
| **F3.3** | 1.0±0.0 | 1.8±0.3 | 2.3±0.1 | 997.2±0.1 | 100.2±0.0 | 20.2±2.8 | 8.3±0.7 | 11.6±0.8 | 2.2±0.1 | 0.2±0.0 | | | 0.1±0.0 | 0.3±0.0 | 21.5±1.1 |
| **F3.4** | 0.8±0.1 | 0.7±0.2 | 1.1±0.4 | 997.2±0.6 | 100.0±0.0 | 20.6±0.4 | 8.7±1.4 | 12.5±2.3 | 1.8±0.1 | 0.2±0.0 | | | 0.1±0.0 | 0.1±0.0 | 18.3±0.7 |
| **F3.5** | 0.8±0.0 | 0.8±0.3 | 1.0±0.4 | 996.6±0.3 | 99.9±0.1 | 20.7±1.8 | 10.3±1.7 | 8.8±0.6 | 1.7±0.0 | 0.2±0.0 | | | 0.1±0.0 | 0.1±0.0 | 17.3±0.5 |
| **F3.6** | 1.1±0.3 | 0.1±0.0 | 1.0±0.3 | 998.1±0.5 | 100.0±0.0 | 20.1±0.4 | 20.3±5.2 | 9.6±0.6 | 1.4±0.0 | 0.2±0.1 | | | 0.0±0.0 | 0.1±0.0 | 13.6±0.3 |
| **B1.1*** | 12.4±1.3 | 8.2±0.4 | 23.8±4.1 | 946.5±7.2 | 99.1±0.3 | 26.5±0.3 | 14.1±0.6 | 23.9±1.0 | 6.3±0.1 | 3.3±0.3 | | | 1.2±0.1 | 5.5±0.8 | 59.5±1.1 |
| **B1.2*** | 9.1±1.0 | 7.1±0.7 | 20.1±3.0 | 957.7±6.1 | 99.4±0.2 | 24.7±0.9 | 17.3±2.0 | 25.3±0.9 | 5.7±0.1 | 2.2±0.2 | | | 1.2±0.0 | 5.0±0.6 | 54.9±1.1 |
| **B1.3** | 3.0±0.2 | 3.3±0.6 | 9.5±3.0 | 974.3±2.8 | 99.0±0.2 | 24.1±1.0 | 12.0±1.3 | 22.4±1.8 | 5.1±0.0 | 0.7±0.0 | | | 0.4±0.1 | 2.0±0.4 | 50.0±0.2 |
| **B1.4** | 2.4±0.1 | 3.5±0.2 | 12.2±1.9 | 983.4±3.5 | 100.2±0.2 | 25.5±1.0 | 13.8±2.8 | 20.9±0.5 | 4.0±0.0 | 0.6±0.1 | | | 0.5±0.1 | 2.5±0.4 | 39.2±0.2 |
| **B1.5** | 1.6±0.1 | 2.3±0.3 | 7.8±0.3 | 990.5±2.8 | 100.2±0.2 | 27.1±1.6 | 13.7±1.8 | 16.0±0.4 | 3.2±0.1 | 0.4±0.0 | | | 0.3±0.1 | 1.2±0.0 | 31.8±0.8 |
| **B1.6** | 0.9±0.1 | 2.1±0.4 | 3.4±0.7 | 996.2±2.6 | 100.3±0.2 | 21.6±1.0 | 9.3±1.0 | 15.2±2.2 | 2.1±0.0 | 0.2±0.0 | | | 0.2±0.0 | 0.5±0.1 | 21.3±0.1 |
| **B2.1** | 5.2±0.4 | 5.5±0.8 | 22.5±3.8 | 957.0±4.4 | 99.0±0.1 | 28.5±0.5 | 21.5±1.6 | 21.6±0.8 | 7.3±0.0 | 1.5±0.1 | | | 1.1±0.1 | 4.8±0.7 | 70.1±0.5 |
| **B2.2** | 5.1±0.1 | 4.7±0.8 | 21.7±1.5 | 967.7±3.8 | 99.9±0.4 | 30.0±1.3 | 20.5±2.2 | 21.8±1.5 | 6.8±0.1 | 1.5±0.0 | | | 0.9±0.1 | 4.7±0.3 | 65.8±0.6 |
| **B2.3** | 4.6±0.1 | 4.9±0.5 | 12.7±2.5 | 970.7±4.1 | 99.3±0.4 | 26.6±1.0 | 19.0±2.3 | 23.9±2.1 | 5.4±0.1 | 1.2±0.1 | | | 0.9±0.0 | 2.9±0.3 | 52.0±0.7 |
| **B2.4** | 4.7±0.3 | 3.1±0.6 | 12.2±3.2 | 975.8±3.6 | 99.6±0.2 | 27.2±0.8 | 23.6±2.7 | 22.4±3.8 | 4.3±0.1 | 1.3±0.1 | | | 0.7±0.0 | 2.4±0.5 | 42.3±0.6 |
| **B2.5** | 4.6±0.2 | 3.3±0.5 | 5.7±2.0 | 980.4±2.3 | 99.4±0.5 | 29.1±0.7 | 25.4±2.5 | 24.7±2.4 | 3.5±0.0 | 1.3±0.0 | | | 0.8±0.0 | 1.3±0.4 | 34.7±0.1 |
| **B2.6*** | 3.2±0.3 | 2.1±0.2 | 5.4±0.6 | 971.7±0.7 | 98.2±0.1 | 27.0±1.3 | 25.6±1.1 | 19.4±1.0 | 2.5±0.0 | 0.9±0.1 | | | 0.5±0.0 | 1.0±0.1 | 24.0±0.2 |
| **B3.1** | 10.8±0.5 | 6.0±0.7 | 22.9±0.1 | 951.9±4.4 | 99.2±0.3 | 26.5±0.8 | 21.4±3.4 | 23.0±1.3 | 8.3±0.0 | 2.8±0.0 | | | 1.2±0.1 | 5.3±0.3 | 79.0±0.2 |
| **B3.2** | 3.9±0.2 | 3.3±0.3 | 9.2±3.4 | 973.1±4.9 | 98.9±0.2 | 30.2±0.6 | 20.0±1.9 | 21.9±2.0 | 5.1±0.1 | 1.2±0.1 | | | 0.6±0.0 | 1.8±0.5 | 49.2±0.9 |
| **B3.3** | 4.0±0.2 | 3.6±0.5 | 9.4±0.7 | 972.7±4.6 | 99.0±0.5 | 31.2±0.4 | 19.8±3.1 | 23.2±1.0 | 4.7±0.0 | 1.3±0.1 | | | 0.7±0.0 | 2.2±0.2 | 45.5±0.1 |
| **B3.4** | 3.1±0.3 | 2.9±0.2 | 8.0±0.8 | 976.9±5.2 | 99.1±0.4 | 26.8±0.6 | 22.3±1.6 | 22.4±1.5 | 4.2±0.0 | 0.8±0.1 | | | 0.6±0.0 | 1.7±0.1 | 41.2±0.5 |
| **B3.5** | 2.2±0.2 | 2.4±0.1 | 6.8±1.1 | 980.7±2.7 | 99.2±0.3 | 30.2±0.7 | 17.2±2.5 | 20.3±3.0 | 3.7±0.1 | 0.7±0.1 | | | 0.4±0.0 | 1.3±0.2 | 35.9±0.6 |
| **B3.6** | 2.0±0.1 | 2.2±0.1 | 5.9±0.2 | 982.1±3.9 | 99.2±0.4 | 23.0±1.2 | 19.7±1.3 | 19.4±1.8 | 3.3±0.0 | 0.5±0.0 | | | 0.4±0.0 | 1.1±0.1 | 32.1±0.1 |
| **B3.7*** | 2.2±0.3 | 2.0±0.1 | 3.9±1.6 | 974.8±1.5 | 98.3±0.2 | 27.6±0.4 | 20.5±1.0 | 19.2±4.2 | 2.3±0.0 | 0.6±0.1 | | | 0.4±0.0 | 0.6±0.1 | 22.3±0.0 |

f-LF: Free-light fraction

o-LF­_10_: Occluded soil organic carbon pool separated after a sonication of 10 J mL^-1^

o-LF­_200_: Occluded soil organic carbon pool separated after a sonication of 200 J mL^-1^

HF: Heavy fraction

mg g^-1^: Extracted material per gram of density fractioned soil, corrected for residual humidity (105°C).

mg C g^-1^: Extracted C per gram of density fractioned soil, corrected for residual humidity (105°C).

Recovery rate: Sum of fraction masses, expressed as % of the fractionated soil mass, corrected for residual humidity (65°C, the same temperature that all fractions were oven dried at).

Suppl. table 2. Chemical composition of the surface (<10 nm depth) of density fractions measured by X-ray photoelectron spectroscopy. Four samples were analysed, two from the **CaCO_3_-free (F2.1 & F2.4)** and two from the **CaCO_3_-bearing** **(B2.1 & B2.4)** sites.

| **Sample** | **Fraction** | **C_1s_** | **O_1s_** | **C:O ratio** | **N_1s_** | **Al_2p_** | **Ca_2p_** | **Cl_2p_** | **Fe_2p3_** | **K_2p_** | **Mg_2s_** | **Na_1s_** | **P_2p_** | **Si_2p_** | **Ti_2p_** | **W_4f_** |
| --- | --- | --- | --- | --- | --- | --- | --- | --- | --- | --- | --- | --- | --- | --- | --- | --- |
|  |  | **%** | **%** |  | **%** | **%** | **%** | **%** | **%** | **%** | **%** | **%** | **%** | **%** | **%** | **%** |
| **F2.1** | f-LF | 51.2 | 35.6 | 1.4 | 0.6 | 3.1 | 0.0 | 2.2 | 0.0 | 0.0 | 0.0 | 0.7 | 0.4 | 6.2 | 0.0 | 0.0 |
|  | o-LF_10_ | 54.6 | 31.6 | 1.7 | 3.7 | 4.3 | 0.1 | 2.4 | 0.0 | 0.0 | 0.0 | 0.3 | 0.0 | 2.3 | 0.1 | 0.6 |
|  | o-LF_200_ | 56.8 | 32.2 | 1.8 | 1.4 | 2.6 | 0.0 | 2.8 | 0.1 | 0.0 | 0.0 | 0.3 | 0.1 | 0.0 | 0.0 | 3.7 |
|  | HF | 45.6 | 34.5 | 1.3 | 2.6 | 2.2 | 0.4 | 8.7 | 1.8 | 0.0 | 0.0 | 0.2 | 0.6 | 3.3 | 0.2 | 0.0 |
| **F2.4** | f-LF | 49.3 | 36.7 | 1.3 | 0.7 | 3.8 | 0.1 | 2.4 | 0.8 | 0.0 | 0.0 | 0.4 | 0.2 | 5.3 | 0.3 | 0.1 |
|  | o-LF_10_ | 51.9 | 33.9 | 1.5 | 0.9 | 6.1 | 0.5 | 4.3 | 0.4 | 0.0 | 0.0 | 0.0 | 0.0 | 1.5 | 0.1 | 0.5 |
|  | o-LF_200_ | 50.9 | 33.6 | 1.5 | 1.7 | 3.8 | 0.3 | 3.2 | 0.2 | 0.0 | 0.0 | 0.1 | 0.0 | 0.9 | 0.4 | 4.9 |
|  | HF | 49.3 | 33.3 | 1.5 | 0.0 | 2.8 | 0.4 | 7.5 | 0.6 | 0.0 | 0.0 | 0.8 | 0.0 | 5.2 | 0.2 | 0.0 |
| **B2.1** | f-LF | 55.8 | 33.0 | 1.7 | 3.0 | 1.5 | 0.3 | 1.5 | 0.0 | 0.0 | 0.0 | 0.2 | 0.0 | 4.7 | 0.0 | 0.0 |
|  | o-LF_10_ | 48.8 | 38.9 | 1.3 | 1.1 | 2.2 | 0.3 | 2.7 | 0.9 | 0.0 | 0.0 | 0.0 | 0.1 | 4.1 | 0.4 | 0.6 |
|  | o-LF_200_ | 48.2 | 34.6 | 1.4 | 2.7 | 4.9 | 1.0 | 3.6 | 0.0 | 0.0 | 0.0 | 0.1 | 0.0 | 2.5 | 0.5 | 1.9 |
|  | HF | 45.8 | 34.1 | 1.3 | 1.7 | 3.0 | 0.5 | 7.2 | 0.9 | 0.0 | 0.0 | 1.5 | 1.0 | 4.2 | 0.3 | 0.0 |
| **B2.4** | f-LF | 51.9 | 37.5 | 1.4 | 0.6 | 2.3 | 0.5 | 3.8 | 1.1 | 0.0 | 0.0 | 0.0 | 1.1 | 0.8 | 0.2 | 0.2 |
|  | o-LF_10_ | 43.7 | 40.2 | 1.1 | 0.0 | 4.3 | 0.5 | 1.6 | 0.4 | 0.0 | 0.0 | 1.8 | 1.1 | 4.9 | 0.0 | 1.5 |
|  | o-LF_200_ | 48.6 | 36.8 | 1.3 | 2.8 | 2.5 | 0.6 | 2.6 | 0.0 | 0.0 | 0.0 | 1.3 | 0.0 | 2.2 | 0.3 | 2.2 |
|  | HF | 51.9 | 30.0 | 1.7 | 0.0 | 2.8 | 0.7 | 9.5 | 1.5 | 0.0 | 0.0 | 0.2 | 0.4 | 2.6 | 0.2 | 0.0 |

Suppl. Table 3. Bulk soil measurements from the **CaCO_3_-free (F1, F2, F3)** and **CaCO_3_-bearing (B1, B2, B3)** profiles, including sample depth intervals, elemental analyser-isotope ratio mass spectrometer measurements (EA-IRMS), and Rock-Eval measurements. EA-IRMS measurements include carbon stable isotope measurements (*δ*^13^C values) and SOC content. Rock-Eval measurements include the total organic carbon (TOC) and mineral carbon (MINC) content, hydrogen index (HI), results from the partitioning of the S2 thermogram (contribution of the A1 to A5 areas under the curve), and the I and R index scores (Matteodo et al., 2018; Sebag et al., 2006). Samples from the CaCO_3_-bearing profiles with the highest carbonate content are marked with an *. More bulk soil properties can be found in Rowley et al. (2020).

| **Sample** | **Depth Intervals** | **EA-IRMS measurements** | | | **Rock-Eval measurements** | | | | | | | | | |  |
| --- | --- | --- | --- | --- | --- | --- | --- | --- | --- | --- | --- | --- | --- | --- | --- |
|  |  | ***δ*^13^C values** | **SOC**  **content** | **TOC** | | **MINC** | **HI** | **S2 thermogram deconvolution** | | | | | | | |
|  | **cm** | **‰** | **%** | **%** | | **%** | **mg HC  g TOC^-1^** | **Contrib.A1** | **Contrib.A2** | **Contrib.A3** | **Contrib.A4** | **Contrib.A5** | **I index** | **R index** | |
| **F1.1** | 0-5 | -26.4 | 4.7 | 5.2 | | 0.9 | 227 | 28.0 | 23.1 | 25.1 | 18.0 | 5.9 | 0.31 | 0.49 | |
| **F1.2** | 5-10 | -25.8 | 3.1 | 3.3 | | 0.7 | 171 | 25.1 | 21.2 | 26.8 | 19.8 | 7.1 | 0.24 | 0.54 | |
| **F1.3** | 10-15 | -25.6 | 2.2 | 2.4 | | 0.5 | 139 | 21.4 | 20.7 | 28.5 | 20.6 | 8.8 | 0.17 | 0.58 | |
| **F1.4** | 15-25 | -25.3 | 1.6 | 1.8 | | 0.5 | 107 | 20.1 | 20.5 | 28.5 | 20.2 | 10.6 | 0.15 | 0.59 | |
| **F1.5** | 25-35 | -25.0 | 1.3 | 1.4 | | 0.4 | 93 | 19.2 | 20.0 | 27.4 | 20.5 | 13.0 | 0.15 | 0.61 | |
| **F1.6** | 35-52 | -24.7 | 0.9 | 1.0 | | 0.4 | 73 | 18.9 | 19.3 | 25.0 | 19.6 | 17.3 | 0.18 | 0.62 | |
| **F2.1** | 0-5 | -26.3 | 5.4 | 5.8 | | 0.7 | 230 | 29.6 | 22.7 | 24.7 | 17.5 | 5.6 | 0.33 | 0.48 | |
| **F2.2** | 5-10 | -26.3 | 2.6 | 2.8 | | 0.5 | 155 | 23.6 | 21.0 | 28.0 | 19.7 | 7.7 | 0.20 | 0.55 | |
| **F2.3** | 10-15 | -25.6 | 2.1 | 2.0 | | 0.2 | 141 | 19.4 | 19.8 | 28.9 | 22.0 | 9.9 | 0.13 | 0.61 | |
| **F2.4** | 15-20 | -25.3 | 1.9 | 1.8 | | 0.2 | 128 | 18.7 | 20.2 | 28.9 | 21.7 | 10.5 | 0.13 | 0.61 | |
| **F2.5** | 20-25 | -25.1 | 1.5 | 1.4 | | 0.3 | 107 | 19.8 | 20.7 | 27.9 | 19.9 | 11.7 | 0.16 | 0.59 | |
| **F2.6** | 25-48 | -25.0 | 0.9 | 0.8 | | 0.1 | 95 | 16.6 | 19.8 | 27.2 | 20.5 | 16.0 | 0.13 | 0.64 | |
| **F3.1** | 0-5 | -26.6 | 5.9 | 6.4 | | 0.5 | 247 | 27.1 | 24.3 | 24.9 | 18.0 | 5.7 | 0.31 | 0.49 | |
| **F3.2** | 5-10 | -25.7 | 3.2 | 3.3 | | 0.5 | 188 | 25.7 | 21.4 | 26.6 | 19.3 | 7.0 | 0.25 | 0.53 | |
| **F3.3** | 10-15 | -25.5 | 2.4 | 2.5 | | 0.4 | 151 | 21.6 | 21.0 | 28.4 | 20.5 | 8.5 | 0.18 | 0.57 | |
| **F3.4** | 15-20 | -25.3 | 2.0 | 1.9 | | 0.2 | 132 | 19.4 | 20.0 | 28.5 | 21.6 | 10.6 | 0.14 | 0.61 | |
| **F3.5** | 20-25 | -24.9 | 1.8 | 1.7 | | 0.2 | 114 | 18.1 | 19.9 | 28.5 | 21.8 | 11.7 | 0.13 | 0.62 | |
| **F3.6** | 25-35 | -24.8 | 1.4 | 1.4 | | 0.3 | 97 | 18.3 | 20.1 | 28.0 | 21.1 | 12.5 | 0.14 | 0.62 | |
| **B1.1*** | 0-5 | -27.4 | 8.0 | 8.2 | | 1.8 | 211 | 23.5 | 26.0 | 25.8 | 18.6 | 6.1 | 0.28 | 0.50 | |
| **B1.2*** | 5-10 | -26.9 | 7.1 | 7.4 | | 1.8 | 208 | 23.7 | 25.2 | 26.1 | 18.8 | 6.2 | 0.27 | 0.51 | |
| **B1.3** | 10-15 | -26.4 | 5.8 | 6.3 | | 1.2 | 190 | 23.0 | 23.8 | 27.1 | 19.8 | 6.3 | 0.24 | 0.53 | |
| **B1.4** | 15-20 | -26.2 | 4.6 | 5.0 | | 1.0 | 172 | 22.5 | 22.7 | 27.8 | 20.3 | 6.8 | 0.21 | 0.55 | |
| **B1.5** | 20-25 | -25.6 | 3.5 | 3.9 | | 0.8 | 153 | 20.5 | 21.8 | 28.7 | 21.6 | 7.5 | 0.17 | 0.58 | |
| **B1.6** | 25-40 | -25.1 | 2.3 | 2.6 | | 0.7 | 126 | 19.8 | 21.8 | 29.1 | 20.9 | 8.5 | 0.16 | 0.58 | |
| **B2.1** | 0-5 | -26.9 | 8.1 | 8.8 | | 1.3 | 224 | 23.5 | 24.1 | 26.0 | 20.2 | 6.3 | 0.26 | 0.52 | |
| **B2.2** | 5-10 | -26.7 | 7.6 | 8.1 | | 1.2 | 207 | 21.5 | 23.5 | 26.9 | 21.9 | 6.3 | 0.22 | 0.55 | |
| **B2.3** | 10-15 | -26.3 | 6.1 | 6.5 | | 1.0 | 196 | 20.6 | 22.3 | 27.9 | 22.6 | 6.6 | 0.19 | 0.57 | |
| **B2.4** | 15-20 | -26.0 | 4.8 | 5.4 | | 0.9 | 174 | 19.2 | 21.0 | 29.7 | 23.6 | 6.5 | 0.13 | 0.60 | |
| **B2.5** | 20-25 | -25.8 | 4.0 | 4.3 | | 0.8 | 155 | 17.2 | 20.4 | 30.9 | 24.5 | 7.1 | 0.09 | 0.62 | |
| **B2.6*** | 25-40 | -25.7 | 2.7 | 3.0 | | 1.6 | 139 | 15.7 | 20.0 | 30.6 | 25.9 | 7.8 | 0.07 | 0.64 | |
| **B3.1** | 0-5 | -26.8 | 9.1 | 9.7 | | 1.3 | 248 | 24.1 | 24.3 | 26.0 | 19.7 | 5.9 | 0.27 | 0.52 | |
| **B3.2** | 5-10 | -26.1 | 5.7 | 5.7 | | 0.6 | 190 | 19.7 | 21.4 | 28.7 | 23.7 | 6.5 | 0.16 | 0.59 | |
| **B3.3** | 10-15 | -26.0 | 5.2 | 5.2 | | 0.6 | 179 | 19.2 | 20.9 | 29.3 | 24.0 | 6.6 | 0.14 | 0.60 | |
| **B3.4** | 15-20 | -25.7 | 4.4 | 4.8 | | 0.6 | 173 | 18.4 | 20.4 | 30.1 | 24.4 | 6.7 | 0.11 | 0.61 | |
| **B3.5** | 20-25 | -25.6 | 3.9 | 3.7 | | 0.4 | 156 | 16.5 | 19.6 | 30.5 | 25.7 | 7.7 | 0.07 | 0.64 | |
| **B3.6** | 25-30 | -25.3 | 3.6 | 3.5 | | 0.3 | 155 | 16.9 | 20.9 | 29.9 | 24.5 | 7.8 | 0.10 | 0.62 | |
| **B3.7*** | 30-37 | -25.1 | 2.4 | 2.4 | | 0.5 | 124 | 17.2 | 20.9 | 29.3 | 22.9 | 9.7 | 0.11 | 0.62 | |

HI index - mg HC g TOC^-1^: milligrams of hydrocarbons pyrolysed per gram of total organic carbon, approximately equivalent to the Van Krevelen H/C ratio.

The OI index (mg O_2_ g TOC^-1^) is not presented as it was erroneously high at the CaCO_3_-free site, which is a known problem when measuring soil samples with a high Fe oxide content (Sebag et al., 2016).

Suppl. Table 4. Stable carbon isotope compositions (*δ*^13^C values) of each individual fraction, separated by density and sonication separated from the **CaCO_3_-free (F1, F2, F3)** and **CaCO_3_-bearing** **(B1, B2, B3)** profiles. There was insufficient material recovered in F1.6 o-LF_10_ for analysis. All figures are mean values of the triplicated fractionation plus or minus the standard error of the mean.

| **Sample** | **Fraction *δ*^13^C values** | | | |
| --- | --- | --- | --- | --- |
|  | **f-LF** | **o-LF_10_** | **o-LF_200_** | **HF** |
|  | **‰** | **‰** | **‰** | **‰** |
| **F1.1** | -27.2±0.1 | -27.8±0.2 | -27.0±0.0 | -25.9±0.0 |
| **F1.2** | -27.4±0.2 | -27.2±0.7 | -26.8±0.2 | -25.8±0.0 |
| **F1.3** | -28.1±0.2 | -27.8±0.3 | -27.4±0.3 | -25.8±0.0 |
| **F1.4** | -27.4±0.1 | -26.7±0.3 | -27.6±0.3 | -25.7±0.0 |
| **F1.5** | -28.0±0.1 | -27.8±0.4 | -28.1±0.1 | -25.5±0.0 |
| **F1.6** | -28.5±0.1 |  | -28.1±0.2 | -25.3±0.0 |
| **F2.1** | -27.7±0.1 | -27.7±0.2 | -27.4±0.2 | -25.7±0.1 |
| **F2.2** | -27.7±0.1 | -28.3±0.4 | -27.5±0.3 | -25.8±0.0 |
| **F2.3** | -27.7±0.1 | -27.8±0.2 | -28.2±0.2 | -25.8±0.0 |
| **F2.4** | -27.0±0.1 | -28.5±0.3 | -28.1±0.2 | -25.8±0.0 |
| **F2.5** | -27.2±0.2 | -28.7±0.9 | -28.1±0.5 | -25.7±0.1 |
| **F2.6** | -27.2±0.4 | -28.1±0.3 | -29.1±0.2 | -25.6±0.0 |
| **F3.1** | -27.2±0.2 | -27.7±0.2 | -26.7±0.0 | -25.8±0.1 |
| **F3.2** | -26.8±0.4 | -28.1±0.3 | -26.9±0.2 | -25.7±0.0 |
| **F3.3** | -26.8±0.6 | -28.8±0.1 | -27.2±0.2 | -25.8±0.0 |
| **F3.4** | -26.4±0.2 | -27.7±0.2 | -27.4±0.2 | -25.6±0.1 |
| **F3.5** | -26.2±0.4 | -27.8±0.0 | -27.6±0.3 | -25.5±0.0 |
| **F3.6** | -25.2±0.2 | -27.3±0.6 | -27.9±0.1 | -25.5±0.0 |
| **B1.1** | -27.2±0.0 | -27.4±0.1 | -26.6±0.0 | -26.5±0.0 |
| **B1.2** | -27.0±0.1 | -27.2±0.3 | -26.4±0.0 | -26.4±0.0 |
| **B1.3** | -26.8±0.1 | -27.1±0.1 | -26.1±0.1 | -26.2±0.1 |
| **B1.4** | -26.3±0.2 | -27.0±0.2 | -25.9±0.0 | -26.0±0.1 |
| **B1.5** | -25.7±0.3 | -27.0±0.1 | -26.0±0.1 | -25.9±0.0 |
| **B1.6** | -26.2±0.2 | -27.4±0.1 | -26.5±0.1 | -25.6±0.0 |
| **B2.1** | -26.6±0.2 | -26.7±0.1 | -26.1±0.0 | -26.1±0.0 |
| **B2.2** | -25.7±0.3 | -26.5±0.2 | -26.0±0.1 | -26.0±0.0 |
| **B2.3** | -25.7±0.1 | -26.1±0.2 | -25.7±0.0 | -26.0±0.1 |
| **B2.4** | -25.2±0.1 | -25.8±0.2 | -25.3±0.1 | -26.0±0.0 |
| **B2.5** | -25.0±0.2 | -25.6±0.1 | -25.3±0.2 | -26.0±0.0 |
| **B2.6** | -25.0±0.0 | -25.5±0.0 | -25.5±0.0 | -26.1±0.0 |
| **B3.1** | -27.5±0.2 | -26.7±0.1 | -26.1±0.0 | -25.9±0.0 |
| **B3.2** | -25.9±0.1 | -26.1±0.0 | -25.7±0.1 | -25.9±0.0 |
| **B3.3** | -24.8±0.2 | -26.0±0.1 | -25.5±0.1 | -25.7±0.1 |
| **B3.4** | -25.4±0.2 | -26.0±0.1 | -25.3±0.0 | -25.9±0.0 |
| **B3.5** | -24.7±0.3 | -26.2±0.3 | -25.5±0.2 | -25.6±0.0 |
| **B3.6** | -25.9±0.2 | -25.6±0.2 | -25.0±0.1 | -25.3±0.0 |
| **B3.7** | -25.9±0.2 | -25.8±0.2 | -25.0±0.3 | -25.3±0.0 |

# Supplementary methods

### Rock-Eval

Rock-Eval measurements were not performed on HCl fumigated bulk samples or the fractions due to the effects of Cl^-^ on the pyrolyser. Thermal decomposition proceeded in two phases. Firstly, samples were pyrolysed in an inert atmosphere (N_2_) between 200-650°C (with intervals of 25°C min^-1^). Secondly, the residual C from the pyrolysis was oxidised in an artificial atmosphere (O_2_/N_2_ mix at 20:80 ratio) between 400-850°C (intervals of 20°C min^-1^). Carbon monoxide (CO) and CO_2_ were measured using an infrared detector, while hydrocarbons (HC) were measured using a flame ionisation detector. Total organic and inorganic carbon were calculated from the HC, CO_2_, and CO produced during the first and second pyrolysis stages using predefined temperature limits (see Behar et al., 2001 for details). The quantity of hydrocarbons produced during the pyrolysis of samples at each temperature interval between 200-650°C (recorded in the “S2” thermogram) was separated into five components representing the area under the curve for fixed temperature ranges, i.e. (A1=200-340°C; A2=340-400°C; A3=400-460°C; A4=460-520°C; A5=520-650°C; Malou et al., 2020). A1 to A5 areas were used to calculate the I and R thermal stability indices (Sebag et al., 2006; Sebag et al., 2016) with Equations 1 & 2, respectively:

$I index= \log_{10} \left( \frac{\left( A1+A2 \right)}{A3} \right)$ (1)

$R index=\frac{A3+A4+A5}{100}$ (2)

### Linear mixed model structures (Rowley et al., 2020)

The effects of the presence and absence of CaCO_3_ (site) on bulk soil variables were investigated using linear mixed models in SAS 9.4^TM^. The estimation method was set to restricted (residual) maximum likelihood. Conditional residuals were plotted against predicted values to evaluate deviations from homoscedasticity and goodness of fit. Residuals were also checked for normality with QQ-plots (Galecki and Burzykowski, 2015). The significance of fixed effects was evaluated using type III F-tests. The denominators’ degrees of freedom were computed using the Satterthwaite adjustment (Satterthwaite, 1946). For significant fixed effects, comparison of means was carried out using t-tests without multiple inference adjustment (Webster, 2007). The alpha level of significance was set at α=0.05 for all tests. All reported means are conditional least-square means ± the standard error of the mean (SEM). Means for profiles are the unweighted average of sampling intervals.

Models included site (CaCO_3_-bearing or free), depth classes and their interaction as fixed effects. Observations were blocked by profile and a different variance was computed for each site since observations from the CaCO_3_-bearing site typically had a higher dispersion than those from the CaCO­_3_­-free site. To account for the autocorrelation of observations within profiles, depth was set as a repeated measure effect with a first-order autoregressive covariance structure. Choice of covariance structure was made based on the Bayesian Information Criteria. Different model structures were used for bulk observations relative to the observations from the density fractions due to the different structure of data in these different datasets.

# References:

Behar, F., Beaumont, V., De B. Penteado, H.L., 2001. Rock-Eval 6 technology: performances and developments. Oil & Gas Science and Technology - Rev. IFP 56(2), 111-134.

ESRI, 2019. World Imagery and national geographic base maps. ESRI, pp. World imagery "Sources: Esri, DigitalGlobe, GeoEye, i-cubed, USDA FSA, USGS, AEX, Getmapping, Aerogrid, IGN, IGP, swisstopo, and the GIS User Community" / National geographic "Sources: National Geographic, Esri, DeLorme, HERE, UNEP-WCMC, USGS, NASA, ESA, METI, NRCAN, GEBCO, NOAA, iPC".

Galecki, A., Burzykowski, T., 2015. Linear mixed-effects models using R: A step-by-step approach. Springer New York, New York.

Malou, O.P., Sebag, D., Moulin, P., Chevallier, T., Badiane-Ndour, N.Y., Thiam, A., Chapuis-Lardy, L., 2020. The Rock-Eval® signature of soil organic carbon in arenosols of the Senegalese groundnut basin. How do agricultural practices matter? Agriculture, Ecosystems & Environment 301, 107030.

Matteodo, M., Grand, S., Sebag, D., Rowley, M.C., Vittoz, P., Verrecchia, E.P., 2018. Decoupling of topsoil and subsoil controls on organic matter dynamics in the Swiss Alps. Geoderma 330, 41-51.

Rowley, M.C., Grand, S., Adatte, T., Verrecchia, E.P., 2020. A cascading influence of calcium carbonate on the biogeochemistry and pedogenic trajectories of subalpine soils, Switzerland. Geoderma 361, 114065.

Satterthwaite, F.E., 1946. An approximate distribution of estimates of variance components. Biometrics Bulletin 2(6), 110-114.

Sebag, D., Disnar, J.R., Guillet, B., Di Giovanni, C., Verrecchia, E.P., Durand, A., 2006. Monitoring organic matter dynamics in soil profiles by 'Rock-Eval pyrolysis': bulk characterization and quantification of degradation. European Journal of Soil Science 57(3), 344-355.

Sebag, D., Verrecchia, E.P., Cécillon, L., Adatte, T., Albrecht, R., Aubert, M., Bureau, F., Cailleau, G., Copard, Y., Decaens, T., Disnar, J.R., Hetényi, M., Nyilas, T., Trombino, L., 2016. Dynamics of soil organic matter based on new Rock-Eval indices. Geoderma 284, 185-203.

Webster, R., 2007. Analysis of variance, inference, multiple comparisons and sampling effects in soil research. European Journal of Soil Science 58(1), 74-82.
